# CD73, a Promising Therapeutic Target of Diclofenac, Promotes Metastasis of Pancreatic Cancer through a Nucleotidase Independent Mechanism

**DOI:** 10.1002/advs.202206335

**Published:** 2022-12-23

**Authors:** Weishuai Liu, Xiaozhou Yu, Yudong Yuan, Yixing Feng, Chao Wu, Chongbiao Huang, Peng Xie, Shengnan Li, Xiaofeng Li, Ziyang Wang, Lisha Qi, Yanan Chen, Lei Shi, Mulin Jun Li, Zhiyong Huang, Bo Tang, Antao Chang, Jihui Hao

**Affiliations:** ^1^ Key Laboratory of Cancer Prevention and Therapy National Clinical Research Center for Cancer Tianjin's Clinical Research Center for Cancer Tianjin Medical University Cancer Institute and Hospital Tianjin 300060 China; ^2^ School of Medicine Nankai University Tianjin 300071 China; ^3^ Tianjin Medical University Tianjin 300070 China; ^4^ Tianjin Institute of Industrial Biotechnology Chinese Academy of Sciences Tianjin 300308 China

**Keywords:** CD73, diclofenac, epithelial‐mesenchymal‐transitions, metastasis, pancreatic ductal adenocarcinoma

## Abstract

CD73, a cell surface‐bound nucleotidase, facilitates extracellular adenosine formation by hydrolyzing 5′‐AMP to adenosine. Several studies have shown that CD73 plays an essential role in immune escape, cell proliferation and tumor angiogenesis, making it an attractive target for cancer therapies. However, there are limited clinical benefits associated with the mainstream enzymatic inhibitors of CD73, suggesting that the mechanism underlying the role of CD73 in tumor progression is more complex than anticipated, and further investigation is necessary. In this study, CD73 is found to overexpress in the cytoplasm of pancreatic ductal adenocarcinoma (PDAC) cells and promotes metastasis in a nucleotidase‐independent manner, which cannot be restrained by the CD73 monoclonal antibodies or small‐molecule enzymatic inhibitors. Furthermore, CD73 promotes the metastasis of PDAC by binding to the E3 ligase TRIM21, competing with the Snail for its binding site. Additionally, a CD73 transcriptional inhibitor, diclofenac, a non‐steroidal anti‐inflammatory drug, is more effective than the CD73 blocking antibody for the treatment of PDAC metastasis. Diclofenac also enhances the therapeutic efficacy of gemcitabine in the spontaneous KPC (LSL‐Kras^G12D/+^, LSL‐Trp53^R172H/+^, and Pdx‐1‐Cre) pancreatic cancer model. Therefore, diclofenac may be an effective anti‐CD73 therapy, when used alone or in combination with gemcitabine‐based chemotherapy regimen, for metastatic PDAC.

## Graphical abstract

CD73 is overexpressed in the cytoplasm of pancreatic cancer cells and promotes metastasis in a nucleotidase‐independent manner. On this basis, a CD73 transcriptional inhibitor diclofenac is then developed, which is more effective than the CD73 blocking antibody for the treatment of metastasis. Moreover, diclofenac also enhances the therapeutic efficacy of gemcitabine both in mouse xenograft and spontaneous pancreatic cancer models.

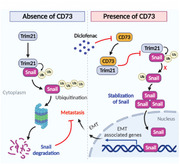

## 1 Introduction

Pancreatic ductal adenocarcinoma (PDAC) is among the most lethal malignancies with dismal prognoses, mainly caused by insidious rapid recurrence or metastasis. Because of the lack of symptoms in the early phase of PDAC, about 80–85% of the patients are diagnosed only after the disease is locally advanced or metastatic.^[^ ^1^ ^]^ Even among those patients who have received curative surgical resection with clear tumor margins (R0), 75% die within 5 years after operation due to recurrence and metastasis.^[^ ^2^ ^]^ Although there has been significant progresses toward understanding tumor metastasis,^[^ ^3^, ^4^ ^]^ the exact molecular mechanisms involved in the invasion and metastatic spread of PDAC remain unclear. We thus sought to determine the key factors driving metastatic PDAC and to develop a therapeutic strategy for its effective treatment.

CD73 (also known as NT5E, ecto‐5‐nucleotidase) catalyzes the conversion of extracellular adenosine monophosphate (AMP) to membrane‐permeable adenosine, a critical response to oxygen deprivation or inflammation. Studies indicate that CD73 is highly expressed in multiple human cancers and is well‐known to promote tumor immune evasion, and thus is considered as an immune checkpoint mediator.^[^ ^5^, ^6^, ^7^ ^]^ Moreover, CD73 has been shown to be a crucial therapeutic target for cancer due to its involvement in tumorigenesis, metastasis, proliferation, apoptosis escape, angiogenesis, and resistance to chemotherapy.^[^ ^8^, ^9^, ^10^, ^11^, ^12^, ^13^, ^14^ ^]^

The number of highly effective inhibitors of CD73 has grown in recent years, including monoclonal antibodies (mAbs) and small molecules that directly inhibit its nucleotidase activity.^[^ ^15^ ^]^ It has been demonstrated in preclinical studies that CD73 blockade may have therapeutic potential when combined with immune checkpoint blocker (ICB)‐based immunotherapy in multiple types of cancer.^[^ ^5^, ^12^, ^15^, ^16^ ^]^ Several CD73 inhibitors are currently being evaluated in clinical trials against various malignant, tumors including PDAC (NCT04148937, NCT04572152, NCT03454451, NCT04672434, NCT03616886, NCT03875573, etc.)^[^ ^17^ ^]^ However, the clinical benefits in some of these trials are limited and controversial, suggesting that the mechanism underling CD73's action in tumor progression is more complex than expected.

Several recent studies have shown that CD73 functions as a prognostic biomarker for PDAC and contributes to its progression by enhancing immune evasion and therapeutic resistance.^[^ ^14^, ^17^, ^18^, ^19^ ^]^ However, the biological role and underlying mechanisms of CD73 in PDAC remain elusive. In the current study, CD73 is predominantly located in the cytoplasm of the PDAC cells. It binds to TRIM21, an E3 ubiquitin ligase, in competition with Snail, to prevent Snail from being degraded by proteasome, thereby promoting epithelial–mesenchymal transition (EMT) and metastatic spread of PDAC. Notably, this pro‐metastatic effect of CD73 in PDAC was independent of its nucleotidase activity. Thus, neither antibodies nor small molecules that inhibit the enzymatic activity of CD73 were effective in preventing this process. Moreover, our study indicated that diclofenac, a non‐steroidal anti‐inflammatory drug (NSAID) commonly used for pain relief, musculoskeletal disorders, migraines, fevers, and acute gout,^[^ ^20^ ^]^ significantly inhibited CD73 transcription. As monotherapy or combined with the first‐line chemotherapeutic agent gemcitabine, diclofenac showed remarkable suppression of PDAC metastasis. Therefore, we have identified a novel non‐canonical mechanism of CD73 mediating PDAC metastasis and developed a new targeting strategy for improving the efficacy of anti‐CD73 treatment.

## 2 Results

### 2.1 CD73 Expression Level Positively Correlates with the Metastatic Potential of PDAC

**Figure 1:**
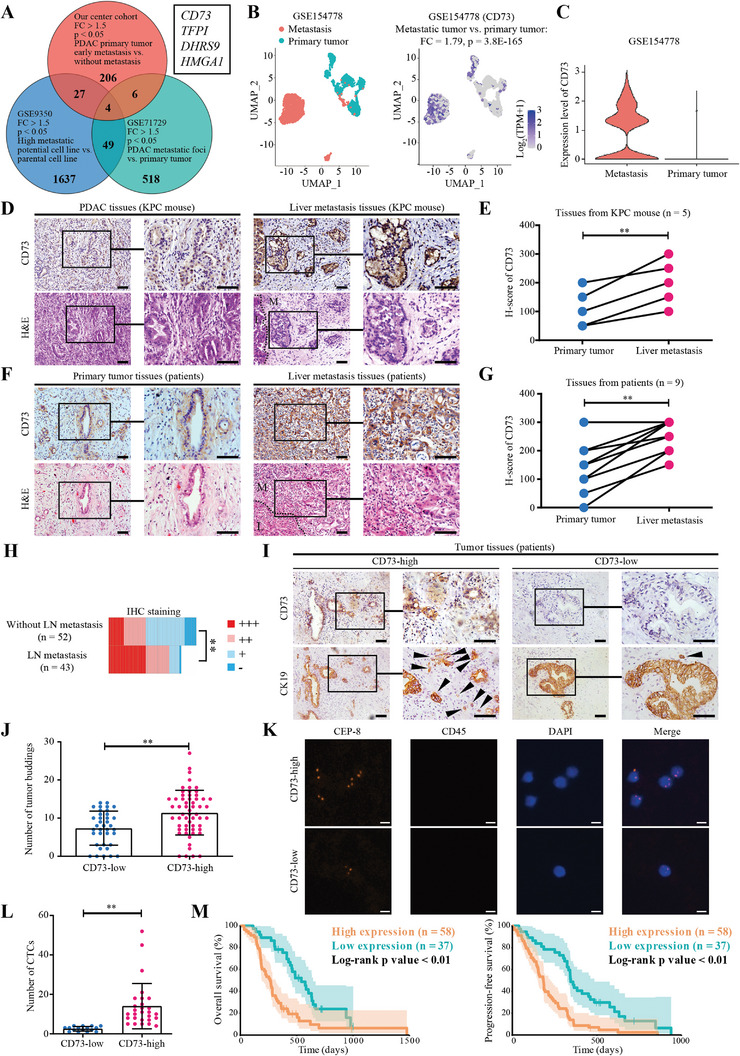
CD73 is highly enriched in the liver metastases and associated with enhanced metastasis and diminished survival of PDAC. A) Scheme of the strategy for identification of target genes involved in PDAC metastasis. Differentially expressed genes (DEGs) with fold changes > 1.5 and *p* < 0.05 in each dataset were enrolled in the Venn diagram. Our center cohort represents the scRNA‐seq data of 2 PDAC patients with early metastasis (recurred within 6 months) in comparison with another 2 patients without metastasis more than 2 years after radical resection in TJMUCIH; GSE9350 is a microarray dataset for comparison of a high metastatic potential daughter cell line to its parental PDAC cell line; GSE71729 is another microarray dataset for comparative analysis between metastases and PDAC primary tumors. B,C) UMAP (B) and violin plots (C) analyzing the expression of CD73 in ductal cell clusters of metastases or PDAC primary tumors from the GSE154778 dataset. D,E) Representative pictures (D) and quantifications of (E) of IHC staining of CD73 in paired primary tumors and liver metastases from KPC mice. H&E staining was performed for morphologic evaluation. *n =* 5. **, *p <* 0.01 in paired *t‐*test. M, metastatic foci; L, normal liver tissue. Scale bar, 100 µm. F,G) Representative pictures (F) and quantifications of (G) of IHC staining of CD73 in paired primary tumors and liver metastases from PDAC patients. *n =* 9. **, *p <* 0.01 in paired *t‐*test. M, metastatic foci; L, normal liver tissue. Scale bar, 100 µm. H) Quantifications of IHC staining of CD73 in primary PDAC tissues with or without LN metastasis at the time of diagnosis. **, *p <* 0.01 in unpaired *t‐*test. LN, lymph node. I,J) Representative pictures (I) and quantifications (J) of tumor budding (stained with CK19) in PDAC tissues with high or low CD73 expression. Arrowheads mark tumor buddings. **, *p <* 0.01 in unpaired *t‐*test. Scale bar, 100 µm. K,L) Representative images (K) and quantifications (L) of circulating tumor cells (CTCs) in 7.5 mL of blood sample from PDAC patients without any treatment before surgery in CD73 high or low expression groups. Data are means ± SD (*n =* 3), **, *p <* 0.01 in unpaired *t‐*test. Scale bar, 5 µm. M) Kaplan–Meier analysis showing that CD73 expression levels correlate with poor overall (left) and progression‐free (right) survival in PDAC patients with radical resection in TJMUCIH. *p* values are from log‐rank tests.

To identify specific transcriptome alterations that might affect PDAC metastasis, we compared the scRNA‐seq data of patients with early metastasis (recurred within 6 months, *n =* 2) to patients without metastasis (*n =* 2) for more than two years after radical resection at Tianjin Medical University Cancer Institute and Hospital (TJMUCIH) (Figure S1A,B, Supporting Information).^[^ ^21^ ^]^ The dataset was converged with two independent public RNA‐Seq datasets which compares high metastatic daughter cells to its parental cells (GSE9350) or metastatic tumor to primary tumor (GSE71729), respectively, to investigate metastasis‐associated genes in PDAC (Figure S1C, Supporting Information). We identified four candidate genes that positively correlated with PDAC metastasis, including CD73, TFPI, DHRS9 and HMGA1 (**Figure** **1**A).

Next, we validated these four genes in another public scRNA‐seq dataset (GSE154778), representing a comparative analysis of the primary PDAC tumor and metastatic tumor (Figure S1D,E, Supporting Information). The results indicated that three of them were enriched in metastatic cells as compared with primary tumor cells, with CD73 being the most enriched gene (Figure 1B,C; Figure S1F,G, Supporting Information). Therefore, we focused on CD73 in the subsequent studies.

We performed immunohistochemical (IHC) staining of CD73 in paired primary tumors and liver metastases from KPC (LSL‐Kras^G12D/+^, LSL‐Trp53^R172H/+^, and Pdx‐1‐Cre) mice or PDAC patients to confirm the sequencing data (Figure S2A, Supporting Information). Notably, the expression level of CD73 was significantly elevated in liver metastases as compared to the primary tumors in both KPC mice (Figure 1D,E) and PDAC patients (Figure 1F,G). Moreover, we analyzed the expression of CD73 in 95 surgical samples from PDAC patients with radical resection in TJMUCIH. Interestingly, CD73 expression in PDAC tumors with lymph node (LN) metastasis was much higher than in those without metastasis at diagnosis (Figure 1H; Figure S2B, Supporting Information). Based on the H‐scores, we then divided these samples into high and low expression groups and determined the role of CD73 in tumor budding and circulating tumor cells (CTCs), which are established indicators for metastasis and prognosis of PDAC.^[^ ^22^, ^23^ ^]^ The CD73 high expression group displayed a higher grade for budding (Figure 1I,J) and more CTCs than the low expression group (Figure 1K,L). Consistently, survival analysis data revealed that the CD73 expression level was negatively associated with overall survival (OS) and progression‐free survival (PFS) of PDAC patients (Figure 1M). Furthermore, we prospectively enrolled a group of patients with similar clinicopathological characteristics from TJMUCIH and followed up on the occurrence of liver metastasis within one year after radical resection. Data revealed a higher incidence of liver metastasis in PDAC patients with high expression of CD73 than in those with low CD73 expression (Figure S2B and Table S1, Supporting Information). Overall, these results demonstrated that the expression of CD73 is associated with enhanced metastasis and diminished survival in PDAC.

### 2.2 CD73 Enhances the Metastasis of PDAC Cells both In Vitro and In Vivo

**Figure 2:**
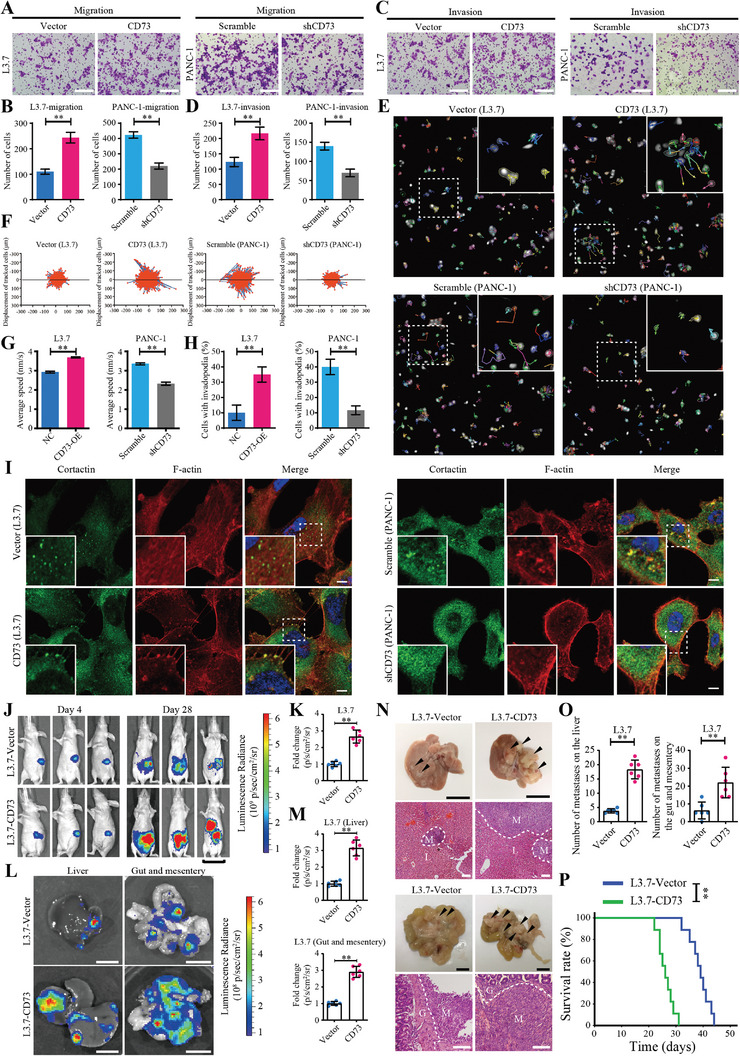
CD73 promotes tumor metastasis in PDAC. A–D) Transwell assays detecting the migration (A,B) and invasion (C,D) of L3.7 cells transduced with CD73 or vector control (Left), or PANC‐1 cells transduced with CD73 shRNA or scrambled shRNA control (Right). Values are means ± SD, *n =* 3. **, *p* < 0.01 in unpaired *t*‐test. Scale bar, 100 µm. E–G) Cell movement analysis of L3.7 cells transduced with CD73 or vector control, or PANC‐1 cells transduced with CD73 shRNA or scrambled shRNA control. Representative dynamic imaging and displacement of tracked cells are shown in (E) and (F), respectively. Quantifications of displacement speed are presented in (G). Values are means ± SD, *n =* 3. **, *p* < 0.01 in unpaired *t*‐test. H,I) Percentage of cells with invadopodia (H) and representative images (I) in L3.7 cells transduced with CD73 or vector control (Left), or PANC‐1 cells transduced with CD73 shRNA or scrambled shRNA control (Right). Invadopodia is identified as co‐localization of cortactin (green) and F‐actin (red) in immunofluorescent staining. Values are means ± SD, *n =* 3. **, *p* < 0.01 in unpaired *t*‐test. Scale bar, 5 µm. J–P) Nude mice were orthotopically injected with firefly luciferase expressing L3.7 (L3.7‐Luc) cells transduced with CD73 or vector control. Tumor burden and metastatic status were monitored by bioluminescent imaging. J,K) Representative bioluminescent images of tumor‐bearing mice on day 4 and day 28 after inoculation (J). Luminescence radiances in abdomen were quantified and then normalized to the vector group (K). Values are means ± SD, *n =* 6. **, *p* < 0.01 in unpaired *t*‐test. Scale bar, 3 cm. L,M) Representative bioluminescent images of metastases in liver, gut, and mesentery from tumor‐bearing mice on day 28 (L). Luminescence radiances in these organs were measured, and then analyzed after normalization to the vector group (M). Values are means ± SD, *n =* 6. **, *p* < 0.01 in unpaired *t*‐test. Scale bar, 1 cm. N–O) Representative images (N) and quantifications (O) of metastatic foci in liver, gut, and mesentery from tumor‐bearing mice on day 28. Arrowheads indicate metastatic foci. Values are means ± SD, *n =* 6. **, *p* < 0.01 in unpaired *t*‐test. Scale bar in the images of metastases, 1 cm; H&E staining, 100 µm. M, metastatic foci; L, normal liver tissue; G, normal gut tissue. P) Kaplan–Meier survival curves of tumor‐bearing mice. *n =* 9. **, *p* < 0.01 in log‐rank test. Data were pooled from three independent experiments.

As CD73 displayed a potential to promote metastasis, we ectopically expressed CD73 in L3.7 and SW1990 cells with low expression, and silenced CD73 in PANC‐1 and BxPC‐3 cells with higher CD73 expression using lentiviral vectors (Figure S3A,B, Supporting Information), and then examined the effect of CD73 on PDAC metastasis. Transwell and wound healing assays indicated that ectopic expression of CD73 dramatically promoted migration and invasion (**Figure** **2**A–D; Figure S3C–H, Supporting Information) in L3.7 and SW1990 cells. In contrast, CD73 knockdown remarkably reduced the migration and invasion of PANC‐1 and BxPC‐3 cells (Figure 2A–D; Figure S3C–H, Supporting Information). Furthermore, cell movement assay using a HCS platform revealed that overexpression of CD73 increased, while silencing of CD73 attenuated the mobility of PDAC cells (Figure 2E–G). Invadopodia formation and extracellular matrix (ECM) degradation are essential steps for tumor cell invasion. Notably, our data demonstrated that CD73 significantly facilitated invadopodia formation (Figure 2H,I) and promoted ECM degradation in PDAC cells (Figure S3I,J, Supporting Information).

To further investigate the role of CD73 in tumor metastasis, we constructed xenograft models by orthotopic injections of firefly luciferase expressing L3.7 cells (L3.7‐Luc) transduced with CD73 or vector control into the pancreas of nude mice. The formation of metastasis was then determined. Bioluminescent imaging data indicated that CD73 overexpression dramatically increased the signal intensity in mouse abdomen (Figure 2J,K) and the liver, gut and mesentery (Figure 2L,M), the specific target organs for PDAC metastatic spread, suggesting that CD73 could enhance the metastatic burden. Consistently, forced expression of CD73 increased the number of metastatic nodules in both liver and gut tissues (Figure 2N,O). Moreover, survival analysis data revealed that mice burdened with CD73 overexpressed tumor displayed a shorter survival time than those with control tumor (Figure 2P). Thus, CD73 plays an essential role in the metastasis of PDAC.

### 2.3 CD73 Promotes PDAC Metastasis in a Nucleotidase‐Independent Manner

**Figure 3:**
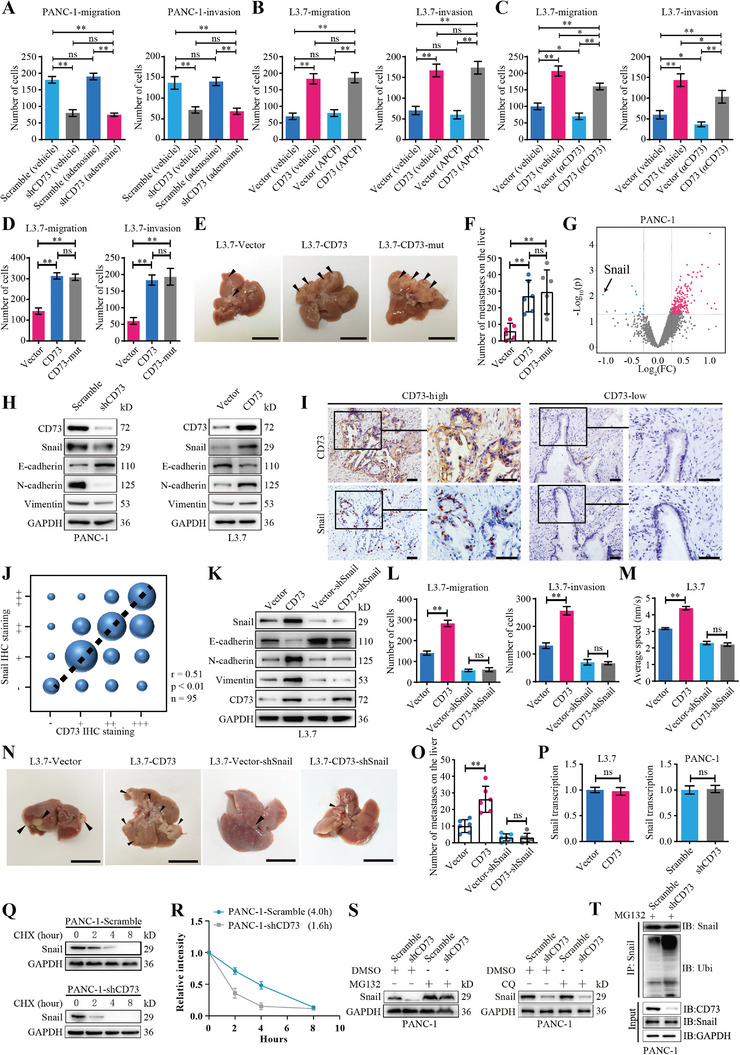
CD73 facilitates PDAC metastasis by upregulating Snail in a manner independent of its nucleotidase activity. A) Quantifications of migration (Left) and invasion (Right) of PANC‐1 cells treated with 1 mm adenosine or vehicle in transwell assays. Values are means ± SD, *n =* 3. ns, not significant, *p >* 0.05; **, *p <* 0.01 using one‐way ANOVA. B,C) Quantifications of migration (Left) and invasion (Right) of L3.7 cells treated with 50 µm APCP (B), or 20 µg mL^−1^ *α*CD73 mAb (C), or vehicle. Values are means ± SD, *n =* 3. ns, not significant, *p >* 0.05; *, *p <* 0.05; **, *p <* 0.01 using one‐way ANOVA. D) Quantifications of migration (Left) and invasion (Right) of L3.7 cells transduced with CD73, a CD73 mutant lacking catalytic activity (CD73‐mut), or vector control. Values are means ± SD, *n =* 3. ns, not significant, *p >* 0.05; **, *p <* 0.01 using one‐way ANOVA. E,F) Nude mice were orthotopically injected with L3.7 cells transduced with CD73, a CD73 mutant, or vector control. Number of metastatic foci on the appearance of liver was measured on day 28 after injection. Arrowheads point to metastatic foci. Values are means ± SD, *n =* 6. ns, not significant, *p >* 0.05; **, *p <* 0.01 using one‐way ANOVA. Scale bar, 1 cm. G) Quantitative proteome (isobaric tags for relative and absolute quantitation, iTRAQ) profiling analysis of PANC‐1 cells transduced with CD73 shRNA or scrambled shRNA control. Differently expressed proteins (*p <* 0.05, fold change > 1.2 or < 0.8) between CD73 knockdown cells and control cells (shCD73 versus control) were presented in the volcano plot. H) Western blot analysis of Snail and EMT markers in PANC‐1 cells transduced with CD73 shRNA or scrambled shRNA control (Left), or L3.7 cells transduced with CD73 or vector control (Right). I,J) Representative IHC staining (I) and spearman rank correlation analysis (J) of CD73 and Snail in PDAC tissues. The bubble size represents the number of patients with indicated staining degree. Scale bar, 100 µm. K–P) Control or CD73 overexpressed L3.7 cells were transduced with Snail shRNA or scramble control, respectively. Western blot (K), transwell (L) and cell movement (M) assays were performed to determine EMT, migration and invasion, and cell mobility of these cells, respectively. Values are means ± SD, *n =* 3. ns, not significant, *p >* 0.05; **, *p <* 0.01 using one‐way ANOVA. N,O) Nude mice were orthotopically injected with indicated L3.7 cells. Number of metastatic foci on the appearance of liver was measured on day 28 after injection. Values are means ± SD, *n =* 6. ns, not significant, *p >* 0.05; **, *p <* 0.01 using one‐way ANOVA. Scale bar, 1 cm. P) qRT‐PCR analysis of CD73 mRNA levels in L3.7 cells transduced with CD73 or vector control (Left), or PANC‐1 cells transduced with CD73 shRNA or scrambled shRNA control (Right). Values are means ± SD, *n =* 3. ns, not significant, *p >* 0.05 in unpaired *t*‐test). Q,R) PANC‐1 cells transduced with CD73 shRNA or scrambled shRNA control were treated with 10 µg mL^−1^ cycloheximide (CHX) for indicated time. The degradation of the Snail protein was measured by western blotting analysis. The level of Snail was quantified after normalization to that of GAPDH using Image J (R). S) Western blotting analysis of PANC‐1 cells transduced with CD73 shRNA or scrambled shRNA control, followed by treatment with MG132 (Left) or chloroquine (CQ) (Right) for 6 h. T) PANC‐1 cells transduced with CD73 shRNA or scrambled shRNA control were treated with MG132 for 6 h. Snail was immunoprecipitated and analyzed by Western blotting to detect ubiquitylated Snail using an anti‐ubiquitin antibody. Data were pooled from three independent experiments.

As CD73 is a nucleotidase responsible for adenosine generation, we investigated whether the enzyme activity of CD73 is required for tumor metastasis. Surprisingly, treatment with adenosine, the metabolic product of CD73, did not restore CD73 knockdown‐mediated loss of migration and invasion ability of PDAC cells (**Figure** **3**A; Figure S4A, Supporting Information). A2AR is the major receptor that mediates the adenosine signaling in tumor cells. Therefore, we treated PDAC cells with SCH58261, a specific small molecule inhibitor of A2AR. Interestingly, A2AR inhibitor did not restrain CD73‐induced migration, invasion (Figure S4B,C, Supporting Information) and cell movement (Figure S4D,E, Supporting Information). mAb (*α*CD73) and 5′‐(*α*,*β*‐ethylene) diphosphate (APCP), a small molecule inhibitor, represent the two major types of CD73 targeting strategies that directly block its nucleotidase activity.^[^ ^19^ ^]^ However, both *α*CD73 mAb and APCP failed to disrupt CD73‐induced migration and invasion in PDAC cells (Figure 3B,C; Figure S4F–H, Supporting Information). These findings suggest that the nucleotidase activity of CD73 is not required for PDAC metastasis.

To confirm these results, we next created a CD73 mutant with no catalytic activity as described previously^[^ ^19^ ^]^ and transduced it into PDAC cells. Consistently, despite a complete loss of nucleosidase activity, the CD73 mutant promoted the migration and invasion of PDAC cells to the same extent as the wild‐type CD73 (Figure 3D; Figure S4I,J, Supporting Information). In the nude mice orthotopic xenograft model, the CD73 mutant and wild‐type CD73 increased the number of liver metastases to similar levels (Figure 3E,F). Thus, our results demonstrate that CD73 enhanced PDAC metastasis primarily through a nucleotidase‐independent mechanism.

### 2.4 CD73 Stabilizes Snail to Enhance PDAC Metastasis

To investigate the mechanism underlying the role of CD73 in tumor metastasis, we performed a quantitative proteomic analysis using a liquid chromatography‐mass spectrometry (LC‐MS) method in PANC‐1 cells. Compared with the scramble control, 23 down‐regulated and 90 upregulated proteins were enriched in cells with CD73 knockdown. Among these proteins, Snail displayed the highest fold change (Figure 3G). Considering its vital role in EMT,^[^ ^24^ ^]^ we chose Snail as the candidate of the downstream effector of CD73 in tumor metastasis.

To verify this hypothesis, we performed immunoblotting to examine the protein level of Snail, and EMT markers E‐cadherin, N‐cadherin and Vimentin in PDAC cells transduced with CD73 or its shRNA. Notably, CD73 overexpression increased the protein level of Snail and mesenchymal markers N‐cadherin and Vimentin but reduced the levels of epithelial marker E‐cadherin (Figure 3H; Figure S5A, Supporting Information). Consistently, IHC staining of CD73 and Snail in serial sections of PDAC samples revealed that the expression level of CD73 correlated positively with that of Snail (Figure 3I,J). We then silenced Snail by shRNA in L3.7 and SW1990 cells transduced with CD73. Interestingly, CD73‐induced migration, invasion, mobility, EMT and liver metastasis of PDAC cells were fully abolished by Snail knockdown, indicating that Snail mediates the pro‐metastatic effect of CD73 (Figure 3K–O; Figure S5B–E, Supporting Information).

In real‐time quantitative PCR (qRT‐PCR) analysis, CD73 did not affect the transcription level of Snail (Figure 3P; Figure S5F, Supporting Information), which was consistent with the results from analysis of The Cancer Genome Atlas (TCGA) data, showing no significant correlation between CD73 and Snail on the mRNA level (Figure S5G, Supporting Information). Thus, CD73 likely upregulates the expression of Snail via post‐transcriptional modification, possibly by regulating its degradation. We thus examined the effect of CD73 on the protein stability of Snail. As expected, overexpression of both wild type and the catalytically inactive mutant of CD73 prolonged the half‐life of Snail protein in L3.7 cells (Figure S5H,I, Supporting Information). In contrast, knockdown of CD73 in PANC‐1 cells reduced both the steady‐state level and the half‐life of the Snail protein (Figure 3Q,R; Figure S5J, Supporting Information). Moreover, CD73 knockdown‐induced downregulation of the Snail was reversed by the treatment of the proteasome inhibitor MG‐132, but not by the lysosome inhibitor chloroquine (CQ) (Figure 3S; Figure S5K,L, Supporting Information). Consistently, CD73 attenuated the level of ubiquitinated Snail in PDAC cells (Figure 3T; Figure S5M, Supporting Information). Therefore, CD73 inhibits ubiquitylation and proteasome‐mediated degradation of Snail to promote PDAC metastasis.

### 2.5 CD73 Interacts with TRIM21 to Prevent Proteasome‐Mediated Degradation of Snail in PDAC Cells

**Figure 4:**
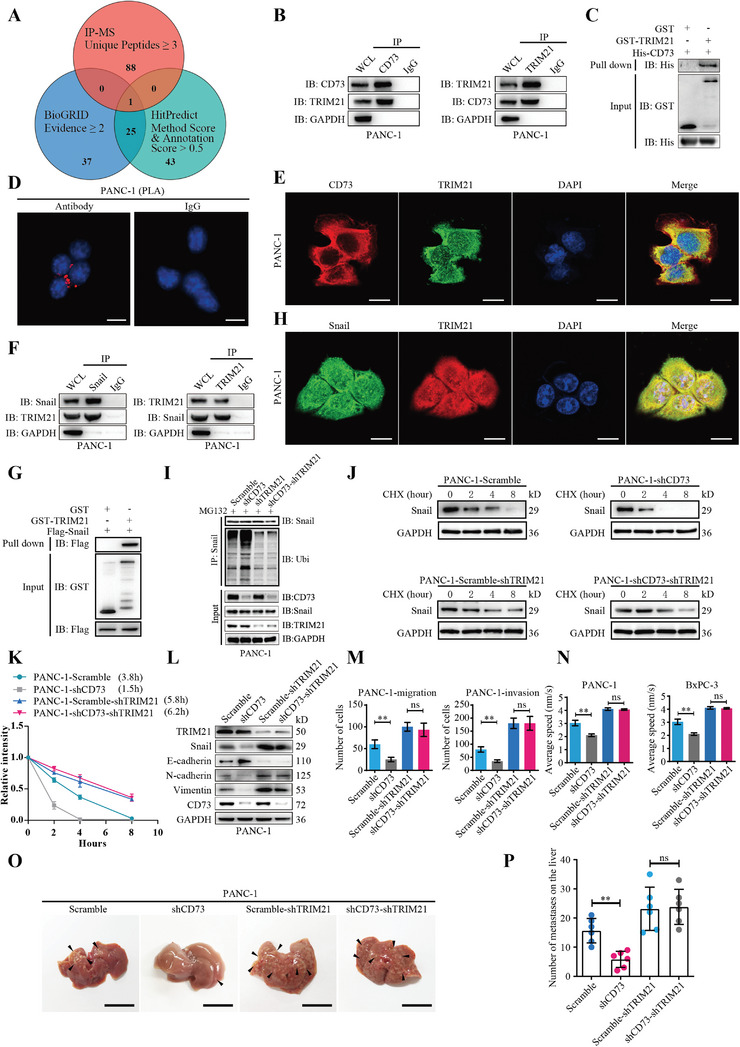
CD73 directly binds to TRIM21 to stabilize Snail in PDAC cells. A) Venn diagram for identification of CD73 binding partners that potentially regulate the degradation of Snail. Flag‐CD73 was immunoprecipitated using an anti‐Flag antibody from L3.7 cells and analyzed by mass spectrometry. A list of potential CD73 binding partners with ≥ 3 unique peptides was then converged with datasets from HitPredict and BioGRID protein interaction databases that are predicted to potentially interact with Snail. B) Co‐IP assays detecting the interaction between endogenous CD73 and TRIM21 in PANC‐1 cells. C) GST pull‐down assays determining the direct binding of recombinant TRIM21 to CD73 in vitro. D) Proximity ligation assay (PLA) examining the binding between CD73 and TRIM21 in PANC‐1 cells in vivo. The red signal represents the formation of CD73‐TRIM21 complex. Mouse IgG with same species and isotype was used as a negative control. Scale bar, 5 µm. E) Colocalization of CD73 (red) and TRIM21 (green) in the cytoplasm of PANC‐1 cells. Scale bar, 5 µm. F) Co‐IP assays showing the interaction between endogenous TRIM21 and Snail in PANC‐1 cells. G) GST pull‐down assays determining the direct binding of recombinant TRIM21 to Snail. H) Colocalization of TRIM21 (red) and Snail (green) in the cytoplasm of PANC‐1 cells. Scale bar, 5 µm. I) Control or CD73 silenced PANC‐1 cells transduced with TRIM21 shRNA or scrambled shRNA control were treated with MG132 for 6 h. Snail was immunoprecipitated and analyzed by Western blotting to detect ubiquitylated Snail using an anti‐ubiquitin antibody. J,K) Control or CD73 silenced PANC‐1 cells transduced with TRIM21 shRNA or scrambled shRNA control were treated with 10 µg mL^−1^ cycloheximide (CHX) for indicated times. The degradation of the Snail protein was measured by western blotting analysis. Representative images were shown in (J). The level of Snail was quantified after normalization to that of GAPDH using Image J (K). L–N) Control or CD73 knockdown PANC‐1 cells were transduced with TRIM21 shRNA or scrambled shRNA control, respectively. Western blotting (L), transwell (M) and cell movement (N) assays were performed to determine EMT, migration and invasion, and cell mobility of these cells, respectively. Values are means ± SD, *n =* 3. ns, not significant, *p >* 0.05; **, *p <* 0.01 using one‐way ANOVA. O,P) Nude mice were orthotopically injected with indicated PANC‐1 cells. Number of metastatic foci on the liver were measured on day 28 after injection. Arrowheads indicate metastatic foci. Values are means ± SD. ns, not significant, *p >* 0.05; **, *p <* 0.01 using one‐way ANOVA. Scale bar, 1 cm. Data were pooled from three independent experiments.

To understand the mechanism underlying the regulation of Snail protein stability by CD73, we conducted the Co‐Immunoprecipitation (Co‐IP) assay for CD73 and Snail. Unexpectedly, Snail did not co‐precipitate with CD73 (Figure S6A, Supporting Information), suggesting that CD73 might regulate the stability of Snail indirectly in PDAC cells. We thus performed immunoprecipitation using an anti‐Flag antibody in L3.7 cells transduced with Flag‐tagged CD73, followed by mass spectrometry (IP‐MS) analysis. A list of candidate proteins that could potentially bind to CD73 was obtained. By referring to the HitPredict (https://www.hitpredict.org/) and BioGRID (https://www.thebiogrid.org/) protein interaction databases, we identified TRIM21, an ubiquitin E3 ligase, as a likely binding partner of both CD73 and Snail (**Figure** **4**A). Therefore, we reason that TRIM21 may act as a link between CD73 and Snail.

Co‐IP assays validated the interaction between both endogenous (Figure 4B) and ectopically expressed (Figure S6B, Supporting Information) CD73 and TRIM21. Moreover, the GST pull‐down assays indicated a direct binding between CD73 and TRIM21 (Figure 4C). In addition, the proximity ligation assay (PLA) illustrated that CD73 physically interacted with TRIM21 in the cytoplasm of PANC‐1 (Figure 4D) and BxPC‐3 cells (Figure S6C, Supporting Information). Immunofluorescence (IF) staining using verified antibodies confirmed an intracellular co‐localization of CD73 and TRIM21 in these two cells (Figure 4E; Figure S6D, Supporting Information).

Co‐IP and GST pull‐down assays also revealed the direct binding of TRIM21 to Snail (Figure 4F,G; Figure S6E, Supporting Information), which was consistent with the IF staining data showing an intracellular co‐localization of these two proteins in PDAC cells (Figure 4H; Figure S6F, Supporting Information). To examine the role of TRIM21 in regulating the expression of Snail, we overexpressed TRIM21 in L3.7 cells with low TRIM21 levels and silenced TRIM21 in PANC‐1 with high TRIM21 levels (Figure S7A, Supporting Information). Notably, TRIM21 reduced Snail expression without influencing the mRNA level, and inhibited EMT, migration and invasion of PDAC cells (Figure S7B–D, Supporting Information). Consistently, IHC staining data indicated that the TRIM21 expression was negatively correlated with Snail expression in PDAC tissues (Figure S7E,F, Supporting Information). In addition, TRIM21 reduced the stability of Snail protein (Figure S7G–J, Supporting Information), which was abolished by the proteasome inhibitor MG132, but not the lysosome inhibitor CQ (Figure S7K,L, Supporting Information). As an E3 ligase, TRIM21 enhanced the ubiquitin level of Snail, leading to its proteasome‐mediated degradation (Figure S7M,N, Supporting Information). Thus, CD73 possibly interacts with TRIM21 to prevent ubiquitylation and proteasome‐mediated degradation of Snail in PDAC cells.

We knocked down TRIM21 in PANC‐1 cells transduced with CD73 shRNA to verify this hypothesis. As expected, CD73 knockdown significantly promoted the ubiquitin level of Snail (Figure 4I), thereby reducing the stability and the level of Snail, which were almost entirely disrupted by the silencing of TRIM21 (Figure 4J,K; Figure S8A, Supporting Information). Furthermore, silencing of TRIM21 restored EMT (Figure 4L), migration and invasion (Figure 4M; Figure S8B, Supporting Information), mobility (Figure 4N; Figure S8C, Supporting Information), and liver metastasis (Figure 4O,P) that were impaired by CD73 knockdown in PDAC cells. Taken together, these results demonstrate that CD73 directly interacts with TRIM21 to prevent ubiquitylation and degradation of Snail, leading to EMT and enhanced metastasis of PDAC cells.

### 2.6 CD73 Directly Binds to TRIM21 in Competition with Snail

**Figure 5:**
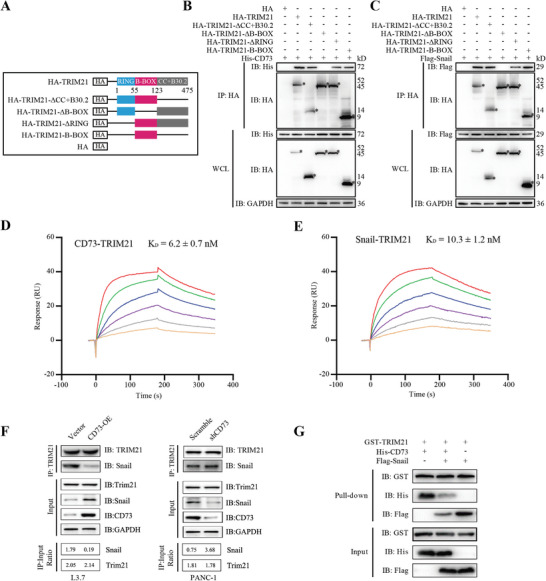
CD73 interrupts the interaction between TRIM21 and Snail. A) A diagram showing the wild‐type and deletion mutatans of TRIM21 used in our study. B,C) HA tagged TRIM21 or its deletion mutatants was co‐transduced with His‐CD73 (B) or Flag‐Snail (C) into HEK‐293T cells, and then immunoprecipitated using an anti‐HA antibody following by Western blotting to determine the domain that mediates the binding of TRIM21 to CD73 or Snail. Asterisks mark the different forms of TRIM21. D,E) Surface plasmon resonance (SPR) assays determining the binding affinity of TRIM21 to CD73 (D) and Snail (E). The chip was immobilized with TRIM21 in advance, and then flowed with gradient concentrations of recombinant CD73 (from top to bottom: 96, 48, 24, 12, 6, and 3 nm) or Snail (from top to bottom: 64, 32, 16, 8, 4, and 2 nm). The K_D_ values are listed. F) TRIM21 was immunoprecipitated from L3.7 cells transduced with CD73 or vector control (Left), or PANC‐1 cells transduced with CD73 shRNA or scrambled shRNA control (Right), and analyzed by western blotting to detect the binding of endogenous TRIM21 to Snail. The levels of Snail and TRIM21 were measured by Image J. The IP:Input ratio was calculated by normalizing the value of immunoprecipitate group to that of input group. G) GST pull‐down assays showing that CD73 directly binds to TRIM21 in competition with Snail. Equal amounts of recombinant proteins (GST‐TRIM21, His‐CD73, or Flag‐Snail) were added to the reaction system, as shown in the input group. Data were pooled from three independent experiments.

To identify the domain that mediates the binding of TRIM21 to CD73 or Snail, we constructed a series of deletion mutations of TRIM21 (**Figure** **5**A) and determined their interactions with CD73 and Snail by Co‐IP assays. Interestingly, the B‐Box domain of TRIM21was responsible for the binding to both CD73 and Snail (Figure 5B,C), suggesting that the binding of CD73 to TRIM21 may spatially compete with Snail.

We next performed a surface plasmon resonance (SPR)‐based assay to examine the binding affinity of TRIM21 to CD73 and Snail, which is indicated by the dissociation constant (K_D_). A lower K_D_ value represents a higher binding affinity.^[^ ^25^ ^]^ Interestingly, the K_D_ value of CD73‐TRIM21 binding is 6.2 ± 0.7 nm (Figure 5D), while that of Snail‐TRIM21 is 10.3 ± 1.2 nm (Figure 5E), indicating that TRIM21 has higher affinity for CD73 than Snail. As CD73 did not affect the expression level and subcellular distribution of TRIM21 (Figure S9A,B, Supporting Information), we reasoned that CD73 might interrupt the binding of TRIM21 to Snail, thereby preventing TRIM21‐mediated ubiquitylation and degradation of Snail.

Co‐IP assays revealed that overexpression of CD73 dramatically reduced the binding of TRIM21 to Snail, while CD73 knockdown enhanced their binding in L3.7 and PANC‐1 cells (Figure 5F). Similarly, Snail also inhibited the binding between CD73 and TRIM21 in these cells (Figure S9C, Supporting Information). Indeed, GST pull‐down assays confirmed that CD73 could directly bind to TRIM21 in competition with Snail in a cell‐free condition (Figure 5G). Together with the previous observations (Figures 3Q–T and 4I–K; Figure S8A, Supporting Information), these findings demonstrate that CD73 directly binds to TRIM21 in competition with Snail, thereby disrupting TRIM21‐mediated ubiquitylation and degradation of Snail, which enhances the expression level of Snail, and subsequently promotes EMT and metastasis of PDAC cells.

### 2.7 Diclofenac Inhibits PDAC Metastasis by Suppressing the Transcription of CD73

Since our data indicate that the pro‐metastatic effect of CD73 in PDAC independent of its canonical nucleotidase activity and cell membrane localization, it cannot be effectively disrupted by the CD73 blocking antibodies or small‐molecule enzymatic inhibitors of CD73. These findings suggested that targeting only the nucleotidase activity may not completely block the tumor‐promoting effect of CD73 in PDAC. Therefore, to develop a better CD73 targeting strategy, we searched for compounds that repress the expression of CD73, aiming to synchronously inhibit both membrane‐bound and intracellular, as well as the nucleotidase‐depend and independent functions of CD73 in tumor cells.

We found three small molecules by referring to a large public dataset analyzing the transcriptome changes before and after treatment with multiple compounds in a hepatic cell line (Genevestigator platform). These molecules, including diclofenac, 4‐(methylnitrosamino)‐1‐(3‐pyridyl)‐1‐butanone (NNK) and omeprazole, remarkably inhibited the transcription level of CD73. NNK is a tobacco‐specific nitrosamine derived from nicotine, identified as a carcinogen,^[^ ^26^ ^]^ making it unsuitable for treatment. Meanwhile, we did not observe any apparent inhibition of CD73 expression in PDAC cells treatment with up to 100 µm omeprazole (Figure S10A, Supporting Information). We thus focused on diclofenac, a widely used NSAID for treating pain and inflammation with a high degree of safety, which is also reported to have an anti‐tumor effect with an unexplained mechanism.^[^ ^20^, ^27^, ^28^ ^]^

**Figure 6:**
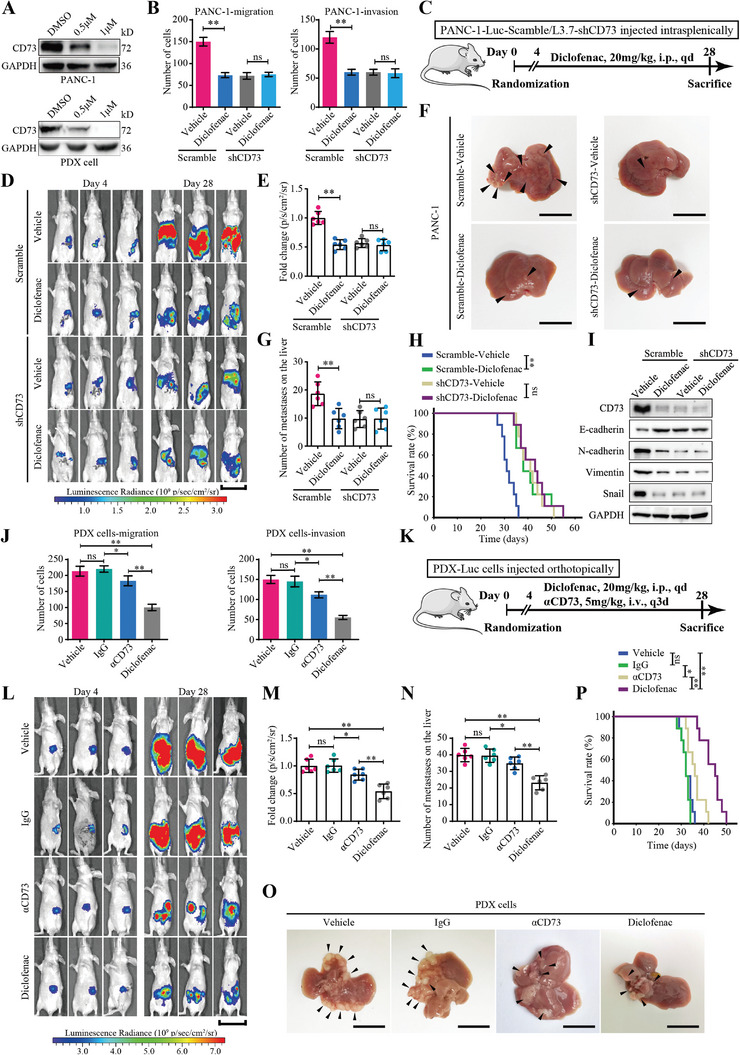
Diclofenac downregulates CD73 and inhibits PDAC metastasis more efficiently than the *α*CD73 mAb. A) Western blot analysis of CD73 in PANC‐1 cells (Top) and primary cells derived from a PDX line (Bottom) treated with 0.5 or 1 µm diclofenac, or DMSO. B) Quantifications of migration (Left) and invasion (Right) of PANC‐1 cells transduced with CD73 shRNA or scrambled shRNA control, followed by treatment with 1 µm diclofenac or vehicle in transwell assays. Values are means ± SD, *n =* 3. ns, not significant, *p >* 0.05; **, *p <* 0.01 in unpaired *t*‐test. C–I) Nude mice were intrasplenically injected with firefly luciferase expressing PANC‐1 cells (PANC‐1‐Luc) transduced with CD73 shRNA or scrambled shRNA control, and treated intraperitoneal daily with 20 mg kg^−1^ of diclofenac or vehicle starting on day 4, as shown in (C). Bioluminescent images of tumor‐bearing mice on day 4 and day 28 after inoculation were measured using an In Vivo Imaging System (D). Luminescence radiances in abdomen were quantified and then analyzed after normalization to the vehicle group (E). Number of metastatic foci on the liver were also measured and quantified on day 28 (F,G). Kaplan–Meier survival curves of tumor‐bearing mice were calculated to determine the therapeutic efficacy of diclofenac (H). The expression levels of CD73 and EMT markers in tumor xenografts in mice were detected by western blotting andanalysis (I). In (E) and (G), all values are means ± SD, *n =* 6. *p* values are calculated using one‐way ANOVA. In (H), *n =* 9. *p* values are from log‐rank tests. ns, not significant, *p >* 0.05; **, *p <* 0.01. J) Quantifications of migration (Left) and invasion (Right) of PDX‐derived cells treated with 20 µg mL^−1^ *α*CD73, or 1 µm diclofenac, or vehicle, or normal IgG control. Values are means ± SD, *n =* 3. ns, not significant, *p >* 0.05; *, *p <* 0.05; **, *p <* 0.01 using one‐way ANOVA. K–P) Nude mice were orthotopically injected with firefly luciferase expressing PDX‐derived cells (PDX‐Luc), and treated intraperitoneal daily with 20 mg kg^−1^ of diclofenac or vehicle, or intravenous with 5 mg kg^−1^ of *α*CD73 mAb every 3 days starting on day 4, as shown in (K). Luminescence radiances of tumor‐bearing mice on day 4 and day 28 were measured (L), and then analyzed after normalization to the vehicle group (M). Number of metastatic foci on the liver were measured and quantified on day 28 (N,O). Kaplan–Meier survival curves of tumor‐bearing mice were calculated to determine the therapeutic efficacy of diclofenac and *α*CD73 mAb (P). In (J) and (N), all values are means ± SD, *n =* 6. *p* values are calculated using one‐way ANOVA. In (P), *n =* 9. *p* values are from log‐rank tests. ns, not significant, *p >* 0.05; **, *p <* 0.01. Scale bars represent 3 cm in (D) and (L), 1 cm in (F) and (O). Data were pooled from three independent experiments.

Consequently, qRT‐PCR and immunoblot data indicated that diclofenac dramatically repressed the mRNA and protein levels of CD73 in a concentration‐dependent manner both in PANC‐1 cells and primary cells derived from a PDX line (**Figure** **6**A; Figure S10B, Supporting Information). With concentrations as low as 1 µm, diclofenac could almost entirely repress the expression of CD73 (Figure 6A; Figure S10B, Supporting Information), thereby increasing the binding between Trim21 and Snail (Figure S10C, Supporting Information). Moreover, diclofenac suppressed migration, invasion and EMT of PDAC cells with CD73 expression, while displaying no additional inhibition in CD73 knockdown cells (Figure 6B; Figure S10D,E, Supporting Information). It is likely that after shRNA‐mediated knockdown (over 90%), the remaining 10% of the CD73 protein is insufficient to confer a detectable metastasis phenotype, and thus metastasis is not further reduced by diclofenac that eliminates the remaining CD73 protein. Therefore, these results suggest that diclofenac inhibits PDAC metastasis by downregulating CD73.

To investigate the therapeutic potential of diclofenac for PDAC metastasis, we constructed xenograft models by intrasplenic injection of firefly luciferase expressing PANC‐1 (PANC‐1‐Luc) cells in nude mice, followed by treatment with diclofenac (Figure 6C). As expected, diclofenac dramatically reduced the number and burden of liver metastases (Figure 6D–G), thereby prolonging the survival time of tumor‐bearing mice (Figure 6H). Meanwhile, immunoblotting data demonstrated that diclofenac suppressed the expression of CD73 and EMT markers in tumor xenografts in mice (Figure 6I). In contrast, CD73 knockdown abrogated the therapeutic effect of diclofenac (Figure 6D–I). Thus, these findings demonstrate that treatment of diclofenac suppresses metastasis by specifically inhibiting the expression of CD73 in PDAC cells.

We also explored the potential mechanism by which diclofenac suppressed the expression of CD73 in PDAC cells. By analyzing The Cancer Genome Atlas (TCGA) database, we identified STAT3 as a downstream effector of diclofenac that regulates the transcription of CD73 (Figure S11A,B and Data files S8, Supporting Information). IHC staining data revealed that the expression level of pSTAT3 was negatively correlated with that of CD73 in PDAC tissues (Figure S11C,D, Supporting Information). Consistently, treatment with the STAT3 antagonist STA‐21 dramatically inhibited CD73 expression on both mRNA and protein levels (Figure S11E,F, Supporting Information). Moreover, as a result of diclofenac inhibiting the phosphorylation‐based activation and nuclear localization of STAT3, we observed transcriptional inhibition of CD73 by inhibiting pSTAT3 binding to the CD73 promoter (Figure S11G–L, Supporting Information). Interestingly, treatment with the STAT3 agonist Colivelin partially restored the effect of diclofenac on CD73 transcription (Figure S11G,L, Supporting Information), suggesting that while suppression of STAT3 at least partly contributes to diclofenac‐induced transcriptional inhibition of CD73, other unknown mechanisms may also be involved.

### 2.8 Diclofenac Displays Better Therapeutic Efficacy than the Mainstream *α*CD73 mAb for the Treatment of Metastatic PDAC

As the *α*CD73 mAb and small‐molecule inhibitor APCP failed to effectively disrupt the stimulating effect of CD73 in metastasis (Figure 3B,C; Figure S4F–H, Supporting Information), diclofenac likely represents a better anti‐CD73 treatment for PDAC than the current CD73 targeting strategies. We thus compared the therapeutic efficacy of diclofenac with *α*CD73 mAb in cell and xenograft models of PDX‐derived cells. We identified the optimal dose of *α*CD73 mAb as 20 µg mL^−1^ in cell culture and 5 mg kg^−1^ body weight in mice models using concentration‐dependence analysis (Figure S12A,B, Supporting Information). Consistently, transwell assays revealed that diclofenac treatment significantly suppressed the migration and invasion of PDX‐derived cells, while *α*CD73 mAb treatment displayed a mild effect in cell culture models (Figure 6J; Figure S12C, Supporting Information). Furthermore, in the orthotopic mice model of PDAC (Figure 6K), diclofenac‐treated mice displayed reduced burden (Figure 6L,M) and number of liver metastases (Figure 6N,O) and exhibited a longer survival time (Figure 6P) than mice treated with *α*CD73 mAb. Altogether, these results demonstrate that diclofenac is a better CD73 targeting agent for treating metastatic PDAC than the mainstream *α*CD73 mAb, which targets the member‐bound and nucleotidase‐dependent activity of CD73.

### 2.9 Diclofenac Enhances the Efficacy of Gemcitabine for the Treatment of Metastatic PDAC

GEM‐based chemotherapy is the most critical treatment choice for PDAC patients after radical resection.^[^ ^29^, ^30^ ^]^ In addition, our previous study indicated that inhibition of CD73 sensitized PDAC cells to GEM treatment.^[^ ^19^ ^]^ Thus, we reason that combination of diclofenac with GEM will likely have great potential to improve the outcome of metastatic PDAC treatment.

**Figure 7:**
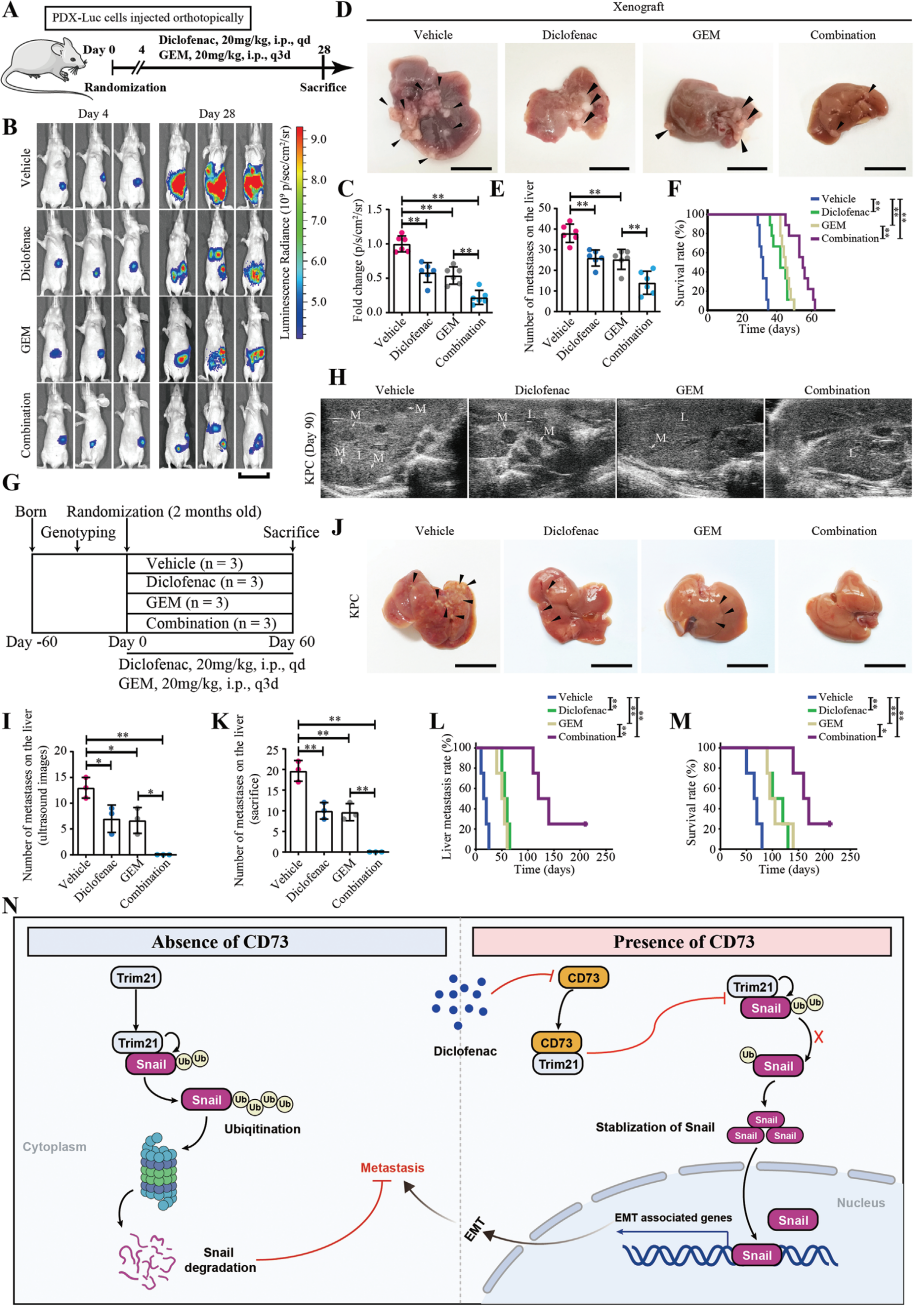
Diclofenac enhances the efficacy of gemcitabine in both xenograft and KPC tumor models. A–F) Nude mice were orthotopically injected with firefly luciferase expressing PDX‐derived cells (PDX‐Luc), and treated intraperitoneal with 20 mg kg^−^ of diclofenac daily, 20 mg kg^−1^ of gemcitabine (GEM) every 3 days, or both, or vehicle starting on day 4 (A). Luminescence radiances of tumor‐bearing mice on day 4 and day 28 were measured (B), and then analyzed after normalization to the vehicle group (C). Number of metastatic foci on the liver were measured and quantified on day 28 (D‐E). Kaplan–Meier survival analysis was used to compare the treatment outcome of different groups (F). In (C) and (E), all values are means ± SD, *n =* 6. *p* values are calculated using one‐way ANOVA. In (F), *n =* 9. *p* values are from log‐rank tests. **, *p <* 0.01. G–M) Two‐month‐old KPC mice with tumors of similar sizes in situ as measured by ultrasound were randomized into 4 groups, and treated intraperitoneally with 20 mg kg^−1^ of diclofenac daily, 20 mg kg^−1^ of GEM every 3 days, or both, or vehicle (G). Liver metastases were measured and quantified by ultrasound at day 90 (H,I). Number of metastatic foci on the liver was determined at the endpoint (J,K). The time when liver metastasis occurs were calculated in (L), and the Kaplan–Meier survival curves were presented in (M). In (I) and (K), all values are means ± SD, *n =* 3. *p* values are calculated using one‐way ANOVA. In (L) and (M), *n =* 4. *p* values are from log‐rank tests. *, *p <* 0.05; **, *p <* 0.01. M, metastatic foci; L, normal liver tissue. Scale bars represent 3 cm in (B), 1 cm in (D) and (J). N) A proposed model for the role of and targeting strategy of CD73 in PDAC metastasis. In the absence of CD73, TRIM21 binds to Snail to induce ubiquitylation and subsequently proteasome dependent degradation of Snail. In the presence of CD73, intracellular CD73 directly binds to TRIM21 in competition with Snail, thereby disrupting TRIM21‐mediated ubiquitylation and degradation of Snail, leading to enhanced EMT and metastasis of PDAC cells, which could be blocked by diclofenac, a transcriptional inhibitor of CD73.

To verify this hypothesis, we constructed orthotopic mice models of PDAC with PDX‐derived cells and treated the mice with diclofenac or GEM alone or in combination (**Figure** **7**A). Notably, our data indicated that GEM or diclofenac monotherapy significantly inhibits metastasis and prolongs the survival time of tumor‐bearing mice (Figure 7B–F). Moreover, the combination of diclofenac with GEM displayed better anti‐tumor efficacy than monotherapies, as indicated by a more significant reduction in the burden (Figure 7B,C) and number of liver metastases (Figure 7D,E) and the even longer survival time (Figure 7F).

Next, we performed a preclinical study using the KPC spontaneous tumor models to confirm these results. Two‐month‐old KPC mice with the same size of the tumor in situ measured by ultrasound detection were randomized into different groups and treated with diclofenac or GEM alone or together (Figure 7G; Figure S12D, Supporting Information). While GEM and diclofenac monotherapies both displayed limited therapeutic efficacy in liver metastasis (Figure 7H–K), no metastatic foci were detected by either ultrasound (Figure 7H) or histology in the liver of mice treated with combination therapy at the endpoint (90 days) (Figure 7J). Meanwhile, in a parallel survival trial, diclofenac combined with GEM delayed the occurrence of liver metastasis and prolonged the survival time of KPC mice to a greater extent than diclofenac or GEM alone (Figure 7L,M), indicating diclofenac could enhance the efficacy of GEM for treatment of metastatic PDAC. Consistently, immunoblotting data confirmed that diclofenac suppressed the expression of CD73 and EMT markers in the tumor tissues of the KPC mice (Figure S12E, Supporting Information). These results demonstrate that diclofenac has great potential to improve the therapeutic efficacy of GEM, and thus diclofenac in combination with GEM is likely an ideal therapeutic strategy for treatment of metastatic PDAC.

## 3 Discussion

From comprehensive analyses of our scRNA‐seq data and publically available datasets, we have identified CD73 as a novel mediator of PDAC metastasis. In the canonical pathway, CD73 is thought to be anchored to the cell‐surface membrane by glycosylphosphatidylinositol (GPI), where it hydrolyzes AMP into adenosine and activates adenosine receptors.^[^ ^5^, ^31^ ^]^ Most previous studies demonstrated that CD73 promoted tumor metastasis as a rate‐limiting enzyme for adenosine production, by signaling through adenosine receptors, A2AR and A2BR.^[^ ^32^, ^33^ ^]^ In several recent studies, CD73 was shown to be present in the cytoplasm^[^ ^19^, ^34^ ^]^ and to function in a nucleotidase‐independent manner. For example, Gao et al.^[^ ^35^ ^]^ reported that CD73 might promote proliferation and migration of human cervical cancer cells by potentiating the EGFR/Akt and VEGF/Akt pathway via an unknown mechanism, which seemed to be independent of its enzyme activity. Wu et al.^[^ ^36^ ^]^ and Mikhailov et al.^[^ ^37^ ^]^ observed that AOPCP, a CD73 enzyme activity inhibitor, could not completely block the effect of CD73 in cell proliferation and TRAIL‐induced apoptosis of cancer cells, respectively, implying an enzyme independent activity of CD73. However, the mechanism underlying these observations remains unclear.^[^ ^38^, ^39^ ^]^ In this study, we provide strong evidence indicating that CD73 is enriched in the cytoplasm of PDAC cells, where it directly binds to the E3 ligase TRIM21 in competition with Snail, disrupting TRIM21‐mediated ubiquitination and degradation of Snail, thereby increasing EMT and metastasis in PDAC cells in a manner independent of its nucleotidase activity.

Despite the high expression level of CD73 in tumor cells, several studies reported that CD73 is also abundant in cancer‐associated fibroblasts (CAFs), resident macrophages, myeloid‐derived suppressor cells (MDSCs), different subsets of T lymphocytes (particularly in Foxp3+ Tregs), and natural killer (NK) cells under specific conditions.^[^ ^40^, ^41^, ^42^, ^43^, ^44^ ^]^ By analyzing a dataset of single‐cell sequencing of PDAC tissues,^[^ ^45^ ^]^ we found that CD73 is highly expressed in some subsets of ductal cells (tumor cells), fibroblasts, endothelial cells and other immune cells, such as macrophages, T cells and B cells (data not shown). Moreover, our data from immunohistochemical staining also indicated that CD73 is mainly expressed in tumor cells, although it is also abundant in some subsets of stromal cells (Figure 1F,I). Thus, these results suggest that, to some extent, tumor cells constitute the prominent CD73‐high population in PDAC.

In an attempt to study the mechanism by which CD73 regulates Snail protein expression, we identified Trim21, a RING finger domain‐containing ubiquitin E3 ligase previously implicated in Snail protein degradation,^[^ ^46^, ^47^ ^]^ as a CD73 interacting protein. Further studies revealed that Trim21 indeed serves as a target of CD73 in the promotion of Snail protein stability. We demonstrate for the first time that CD73 directly binds to TRIM21 and competes with Snail for its biding site, resulting in reduced ubiquitylation and stabilization of Snail. Our data indicate that Trim21 is both necessary and sufficient for Snail protein ubiquitination and degradation and thus is a key regulator of Snail protein expression. However, despite that Trim21 directly binds to Snail, we cannot rule out the possibility that Trim21 indirectly mediates Snail ubiquitination and degradation. We are currently performing cell‐free ubiquitination assay to determine whether Trim21 directly catalyzes the ubiquitination of Snail. Nevertheless, even in the unlikely scenario that Snail is not a direct TRIM21 substrate, our conclusion that CD73 increases Snail protein stability and promotes PDAC metastasis by disrupting Trim21‐mediated Snail ubiquitination and degradation is still valid.

CD73 inhibitors, including mAbs and small‐molecule enzymatic inhibitors, are now available with potency at various concentrations. Currently, they are undergoing clinical trials against malignant tumors based on their ability to directly inhibit the nucleotidase activity of CD73.^[^ ^15^, ^48^ ^]^ As a result of minimal overall clinical efficacy, several trials (e.g., NCT03954704 and NCT04262388 in clinicaltrials.gov) involving CD73 blocking antibodies or small molecule inhibitors for advanced solid tumors have been discontinued. Interestingly, our data indicate that neither the blocking antibody nor the small molecule inhibitor could effectively disrupt CD73‐induced metastasis of PDAC cells. Consequently, targeting the nucleotidase activity only may not be enough to fully block the tumor‐promoting effect of CD73 in PDAC.^[^ ^13^, ^49^ ^]^ Moreover, anti‐CD73 mAb treatment may also result in the internalization of CD73, which may further increase the concentration of intracellular CD73.^[^ ^50^ ^]^ As a result, an optimal CD73 targeting strategy should also consider the intracellular and nucleotidase‐independent functions of CD73. Therefore, suppressing the expression or inducing the degradation of the total CD73 protein may be the most effective way for increasing the anti‐CD73 efficacy.

Traditional de novo drug discovery is cost‐intensive and time‐consuming, and has a high failure rate.^[^ ^51^ ^]^ Instead, a drug repurposing strategy involves identifying new uses for approved drugs beyond their original therapeutic indication. In this study, we identified diclofenac, a NSAID widely used to treat pain, fever and inflammation,^[^ ^28^ ^]^ as an effective transcriptional inhibitor of CD73. Based on epidemiological evidence, NSAIDs, particularly aspirin, decrease the risk and mortality of a wide variety of cancers.^[^ ^52^ ^]^ Moreover, we found that diclofenac shows a therapeutic effect for metastatic PDAC, which is significantly greater than the mainstream *α*CD73 mAb, suggesting that diclofenac may be optimized for CD‐73‐targeting therapies in cancer. These results are consistent with recent reports suggesting that diclofenac has an anti‐tumor effect in multiple cancers.^[^ ^20^, ^27^, ^28^ ^]^ Additionally, pain and inflammation are among the most common symptoms and complications in PDAC patients,^[^ ^53^, ^54^ ^]^ often treated with a low dose of diclofenac.^[^ ^55^ ^]^ Thus, diclofenac may represent a potential treatment option for metastatic PDAC.

Similar to other NSAIDs, diclofenac inhibits prostaglandin synthesis by inhibiting cyclooxygenase (COX)‐1 and COX‐2.^[^ ^56^ ^]^ Diclofenac inhibits COX‐2 with greater potency than COX‐1.^[^ ^57^ ^]^ However, one study showed that NSAIDs do not require the presence of COX‐2 to treat cancer;^[^ ^58^ ^]^ thus, we believe that diclofenac may suppress CD73 transcription in a manner independent of COX‐2. In fact, our data indicated that diclofenac reduced the phosphorylation‐based activation of STAT3, thereby inhibiting pSTAT3‐induced transcription of CD73 in PDAC cells. Interestingly, treatment with the STAT3 agonist just partially restored the inhibitory effect of diclofenac on CD73 transcription, suggesting that other mechanisms may also be involved in diclofenac‐induced transcriptional inhibition of CD73, which requires further investigation.

Considering its crucial role in modulating the tumor microenvironment, particularly in immune evasion,^[^ ^59^, ^60^ ^]^ CD73 has emerged as an important candidate for cancer immunotherapy.^[^ ^61^ ^]^ In fact, CD73 blockade has demonstrated therapeutic effects as a monotherapy or when combined with immune checkpoint blocker (ICB)‐based immunotherapy in multiple types of cancer.^[^ ^15^, ^16^ ^]^ Therefore, further research is required to determine the roles of diclofenac in regulating immune responses and interfering with stromal cells, and how these mechanisms contribute to diclofenac's anti‐tumor efficacy in metastatic PDAC.

Since PDAC is a highly lethal malignancy, a single treatment strategy is unlikely to produce optimal outcomes; and therefore, combination therapy may maximize the anti‐tumor benefits of drugs. Based on our findings, diclofenac has great potential to improve the therapeutic efficacy of gemcitabine, an agent used as the first‐line treatment of PDAC,^[^ ^62^, ^63^ ^]^ in both xenograft and spontaneous pancreatic cancer models. Thus, diclofenac combined with gemcitabine may greatly benefit patients suffering from metastatic PDAC.

## 4 Experimental Section

### Cell Culture

The human PDAC cell lines PANC‐1, BxPC‐3, MIA PaCa‐2 and SW1990, and human embryonic kidney cell line HEK‐293T, were purchased from the American Type Culture Collection (ATCC). The human PDAC cell line L3.7 was a gift from Prof. Keping Xie (MD Anderson Cancer Center, Houston, TX). The authenticity of the cells was verified using short tandem repeat (STR) profiling, and mycoplasma contamination was ruled out. PANC‐1, MIA PaCa‐2, and HEK‐293T cells were cultured in Dulbecco's modified eagle medium (DMEM) with 10% fetal bovine serum (FBS) (Gibco). BxPC‐3, L3.7, and SW1990 cells were maintained in Roswell Park Memorial Institute Medium (RPMI)‐1640 supplemented with 10% FBS. All cells were cultured at 37 °C in a humidified incubator containing 5% CO_2_.

### Human Sample Collection

Human tissue samples were retrospectively collected from PDAC patients with radical resection under the National Comprehensive Cancer Network (NCCN) guidelines from July 2011 to April 2014 at TJMUCIH. All patients signed an informed consent approved by the Ethics Committee of the TJMUCIH.

### Histological Analysis

5 µm paraffin‐embedded sections were prepared and stained with hematoxylin and eosin for morphologic evaluation and tumor confirmation. IHC analyses of CD73, CK19, TRIM21 and Snail were performed using the Envision Kit (Dako) as described,^[^ ^64^, ^65^ ^]^ with modifications for each antibody (Table S2, Supporting Information). Images were obtained with KAYENCE BZ‐X810 All‐in‐One Fluorescence Microscope (Japan) and examined by a pathologist.

IHC staining was evaluated blindly by two independent people unaware of the patients’ clinical information. The intensity of staining was graded into negative (score 0), low (score 1), medium (score 2), and high (score 3). For positive staining rates, a positive cell percentage of 0% was scored as 0, 1–25% as 1, 26–50% as 2, and more than 50% as 3. The multiplication of positive rate and intensity score or the H‐score method was used to determine the expression levels of target genes. Accordingly, the degree of expression was divided into four grades: 0 for negative (−); 1 to 2 for low staining (+); 3 to 5 for medium staining (++); 6 to 9 for high staining (+++).^[^ ^64^ ^]^ The H‐score was calculated using the following formula: [1 × (percentage cells of score 1) +2 × (percentage cells of score 2) + 3 × (percentage cells of score 3)].^[^ ^19^ ^]^

### Immunofluorescence Staining

Cells were seeded on cover glass in a 24‐well plate and cultured for 24–48 h. After washing with PBS, cells were fixed with 4% formaldehyde in PBS for 10 min and permeabilized with 0.5% Triton‐X100 in PBS for 5 min. Cells were then washed 3 times with 0.1% PBS‐Tween (PBST), blocked with 3% BSA in PBST for 1 h, washed 3 times with 0.1% PBST and incubated with indicated primary antibody overnight at 4 °C (Table S2, Supporting Information). Cells were then washed 3 times with 0.1% PBST and incubated with indicated secondary antibodies (ThermoFisher, USA) in 3% BSA in the dark for 1 h at room temperature. After washing 3 times with 0.1% PBST, cells were mounted with Antifade Mounting Medium with DAPI (Vector laboratories) and sealed with clear nail polish. Images were captured with a fluorescence microscope (ZEISS, Germany).

### Quantification of Tumor Budding

Evaluation of tumor budding was performed as described by the International Tumor Budding Consensus Conference (ITBCC).^[^ ^66^ ^]^ Briefly, tumor sections were stained with CK19 to mark the PDAC cells, and screened for the most relevant tumor field at low magnification (40×), and then the hotspots were analyzed at high magnification (40×). Tumor budding was defined as the presence of de‐differentiated single cells or small clusters of up to 5 cells in the tumor stroma at the invasive front. Five random fields (400× magnification) per slide were quantified blindly by two independent pathologists.

### Circulating Tumor Cells (CTCs) Determination

7.5 mL blood sample was collected from PDAC patients without any treatment before surgery. The isolation and identification of CTCs were performed by using the negative enrichment and immunofluorescence in situ hybridization (NE‐iFISH) as described before.^[^ ^67^ ^]^ Images were obtained with a fluorescence microscope (ZEISS, Germany) and quantitated in a double‐blinded manner.

### Migration and Invasion Assays

PDAC cells were resuspended in a 200 µL serum‐free medium, seeded into an 8 µm‐pore chamber with or without matrix gel (BD Biosciences, USA) for invasion or migration assays, respectively, and then inserted into 24‐well plates containing 500 µL complete medium and cultured for the indicated time. Chambers were then fixed in 70% ethanol and stained with 0.05% crystal violet for 2 h. After clearing the cells from the inner surface with a swab, membranes were carefully removed and mounted on slides for visualization with KAYENCE BZ‐X810 All‐in‐One Microscope (Japan). The number of invasive cells in five random fields per slide was counted.

### Wound‐Healing Assay

Cells were cultured in 6‐well plates for 24 hours until they were 90–95% confluent. A sterile 10 µL pipet tip was used to create a straight scratch in the cell monolayer. After washing with PBS to remove the floating cells, the plate was incubated in a basic medium containing 1% FBS. Images were captured at an indicated time point by a microscope (Olympus, Japan).

### Gelatin Degradation and Invadopodia Formation Assay

The matrix degradation assay was conducted using a commercial kit according to the manufacturer's protocols (Sigma‐Aldrich, USA). For invadopodia formation assay, cells were subjected to an immunofluorescence analysis of cortactin and F‐actin/phalloidin. Invadopodium was indicated as co‐expression of cortactin and F‐actin. Co‐expression of cortactin and F‐actin indicated invadopodia. Five random fields per sample were quantified.

### Cell Movement Assay

4000 cells were seeded in a 24‐well plate for 24 h and then automatically tracked for 16 h by the Cell Insight CX7 High‐Content Screening (HCS) Platform (ThermoFisher, USA). Data were analyzed using the Harmony Software (PerkinElmer, USA).

### Immunoblot

PDAC cells were collected and lysed in sodium dodecyl sulfate (SDS) lysis buffer supplemented with 1 × protease (Sigma‐Aldrich, USA) and phosphatase (Bimake, USA) inhibitors for 30 min on ice and then centrifuged at full speed for 10 min at 4 °C. The supernatants were collected and subjected to SDS‐PAGE followed by immunoblotting.^[^ ^68^ ^]^ Signals were detected using the Immobilon Western HRP Substrate (Millipore, USA) and captured by the Tanon 4800 Imaging System (China). The antibodies used for immunoblotting are listed in Table S2, Supporting Information.

### RNA Isolation and qRT‐PCR

Total RNA was extracted using TRIzol reagent (Invitrogen, USA), transcribed to cDNA using a commercial reverse transcription supermix (Bimake, USA), and quantified by qRT‐PCR using SYBR Green qPCR Master Mix (Bimake, USA) in Bio‐Rad CFX96 following manufacturer's protocols. *β*‐actin was used as an internal control to normalize the mRNA level for each gene. PCR primers are listed in Table S3, Supporting Information.

### Vector Construction and Lentivirus‐Based Gene Transduction

For gene expression, the CDS of the target gene was amplified by reverse‐transcription PCR and then inserted into the pLV‐EF1*α*‐MCS‐IRES‐Puro/Neo cloning vector (Biosettia, USA) with indicated restriction enzymes. For gene silencing, single‐stranded shRNA was annealed and then ligated to the pLV‐H1‐EF1*α*‐Puro Vector (Biosettia, USA) according to the manufacturer's protocols. Recombinant lentiviruses were packaged and transduced into cells as described before.^[^ ^69^ ^]^ shRNA sequences are listed in Table S3, Supporting Information.

### Co‐Immunoprecipitation (Co‐IP), Liquid Chromatography (LC)‐Mass Spectrometry (MS)/MS, and Glutathione S‐transferase (GST) Pull‐Down

Cells were lysed in the IP lysis buffer (ThermoFisher, USA) supplemented with 1 × protease and phosphatase inhibitors for the Co‐IP assays. Protein extracts were incubated with indicated antibodies (Table S2, Supporting Information) overnight at 4 °C in a rotator, captured by Protein A/G PLUS‐Agarose (Santa Cruz Biotechnology, USA) and then subjected to SDS‐PAGE followed by immunoblotting as described above, or LC‐MS/MS analysis using Orbitrap Elite Hybrid Mass Spectrometer on a fee‐for‐service basis (Wayenbio, China). The polypeptide sequence was identified using Proteome Discoverer (ThermoFisher, USA) and Mascot server (Matrix Science, UK) (Table S4, Supporting Information).

GST pull‐down was performed using the Pierce GST Protein Interaction Pull Down Kit (ThermoFisher, USA) following the manufacturer's instructions. Briefly, GST or GST‐TRIM21 was conjugated to glutathione agarose, then incubated with recombinant CD73 or Snail. The immobilized proteins were then eluted and subjected to immunoblot (Table S4, Supporting Information).

### Duolink Assay

Duolink assay was performed using the Duolink In Situ Red Starter Kit Mouse/Rabbit (Merck, USA), following the manufacturer's instructions. Images were captured using a fluorescence microscope (ZEISS, Germany).

### Surface Plasmon Resonance (SPR) Assay

Surface plasmon resonance (SPR) assay was performed on a Biacore 8K system (GE Healthcare, USA) using a CM5 sensor chip according to the manufacturer's instructions. Data were acquired and analyzed by the Bioevaluation software (GE Healthcare).

### Proteomic Analysis

Proteomic analysis was performed using the Thermo Scientific LC‐MS system according to the manufacturer's instructions. For each sample, 150 µg protein sample was digested by 3 µg trypsin in 500 µL of 50 mm triethylammonium bicarbonate (TEAB) buffer, labeled with an isobaric tags for relative and absolute quantitation (iTRAQ) Reagent‐Multiplex Buffer Kit (AB SCIEX, Germany), separated using the EASY‐nLC1200 LC system (ThermoFisher, USA) with C18 column (C18, 2 µm, 100 Å, 75 µm × 25 cm, nanoViper), and then subjected to the Orbitrap Exploris 480 Mass Spectrometry (ThermoFisher, USA) for quantitative proteomics. The raw files delivered by the mass spectrometer were analyzed using the Proteome Discoverer 3.0 search engine (ThermoFisher, USA). Proteins with at least one unique peptide and a false discovery rate of less than 1% were included. Isobaric tags for relative and absolute quantitation (iTRAQ) 8‐plex was used for quantification.

### Animal Models

5‐week‐old female BALB/c nude mice (Homozygous) were purchased from Beijing HFK Bioscience CO., LTD (China). The KPC (LSL‐Kras^G12D/+^, LSL‐Trp53^R172H/+^ and Pdx‐1‐Cre) mouse models were kindly provided by Dr. Xueli Bai (The First Affiliated Hospital of Zhejiang University, China) as a gift. All mice were maintained under pathogen‐free conditions following the guidelines for laboratory animals of TJMUCIH. All studies were approved by the Ethics Committee of TJMUCIH.

Primary cells derived from a PDX (patient‐derived xenograft) line were obtained as described before.^[^ ^70^ ^]^ 6‐week‐old female BALB/c nude mice were orthotopically or intrasplenically injected with 1 × 10^6^ of L3.7, PANC‐1 or PDX‐derived cells transduced with firefly luciferase, and treated intravenous with 5 mg kg^−1^ *α*CD73 mAb every 3 days, or intraperitoneal with 20 mg kg^−1^ of diclofenac daily, 20 mg kg^−1^ of gemcitabine (GEM) every 3 days, or combination. Tumor burden and metastatic status were measured every week using an in vivo Imaging System (IVIS) (PerkinElmer, USA). Tumor, liver, gut and mesentery tissues were harvested at the end of the studies, and subjected to the indicated analysis.

Two‐month‐old KPC mice with tumors of similar sizes in situ as measured by ultrasound detection were randomized into 4 groups, and treated intraperitoneal with 20 mg kg^−1^ of diclofenac daily, 20 mg kg^−1^ of GEM every 3 days, or both, or vehicle (Table S4, Supporting Information). Liver metastases were measured and quantified by ultrasound every week. Tumor and liver tissues were harvested at the end of the studies, and subjected to the indicated analysis.

### Data Analysis of Single‐Cell RNA Sequencing (scRNA‐seq)

The gene expression matrix of scRNA‐seq was further processed using R software (v3.6.0). Principal component analysis, Uniform Manifold Approximation and Projection (UMAP), and hierarchical clustering of scRNA‐seq data were administered by using the Seurat package. The Wilcoxon test determined differentially expressed genes (DRGs). The clusters of cell‐type assignments were completed with a set of known marker genes for each cell type as reported.^[^ ^71^, ^72^, ^73^ ^]^ In hierarchical clustering, the log2‐transformed values of gene expression were converted to global Z‐Scores. In the cohort from the authors’ center, two patients (P05 and P14) with early recurrence (within 6 months after radical resection) and two patients (P01 and P03) without metastasis (more than 2 years after radical resection) were included in the analysis.

### Statistical Analysis

All statistical analyses were done using the SPSS version 19 (IBM, USA). The statistical tests, such as Student's *t*‐test, Mann–Whitney U test or ANOVA, used to determine significant differences of variables between groups were indicated in the figure legends. The Kaplan–Meier method was preformed to compare survival between groups, and Log‐rank tests were applied to analyze risk factors that affected prognosis. Spearman or Pearson correlation coefficients were used to determine the correlation between variables. Data were expressed as the means ± SD (standard deviation) unless otherwise stated. *p* value < 0.05 was considered significant.

## Conflict of Interest

The authors declare no conflict of interest.

## Supporting information

Supporting Information

Supporting Information xlsx

## Data Availability

The data that support the findings of this study are available in the supplementary material of this article.
