# Supplementary material for: CD73, a Promising Therapeutic Target of Diclofenac, Promotes Metastasis of Pancreatic Cancer through a Nucleotidase Independent Mechanism
Source: Adv Sci (Weinh). 2022 Dec 23;10(6):2206335. doi: 10.1002/advs.202206335 (PMC9951332; doi:10.1002/advs.202206335)
Supplement: Supplementary file 1 — Supporting Information [file ADVS-10-2206335-s001.pdf]

Supporting Information

Title CD73, A Promising Therapeutic Target of Diclofenac, Promotes Metastasis of Pancreatic Cancer through A Nucleotidase Independent Mechanism

W. Liu, X. Yu, Y. Yuan, Y. Feng, C. Wu, C. Huang, P. Xie, S. Li, X. Li, Z. Wang, L. Qi, B. Tang, A. Chang* and J. Hao**

Content

Supplementary Figures (Figure S1-S12)
Supplementary Tables (Table S1-S4)
Supplementary Materials and Methods

Supplementary Figures

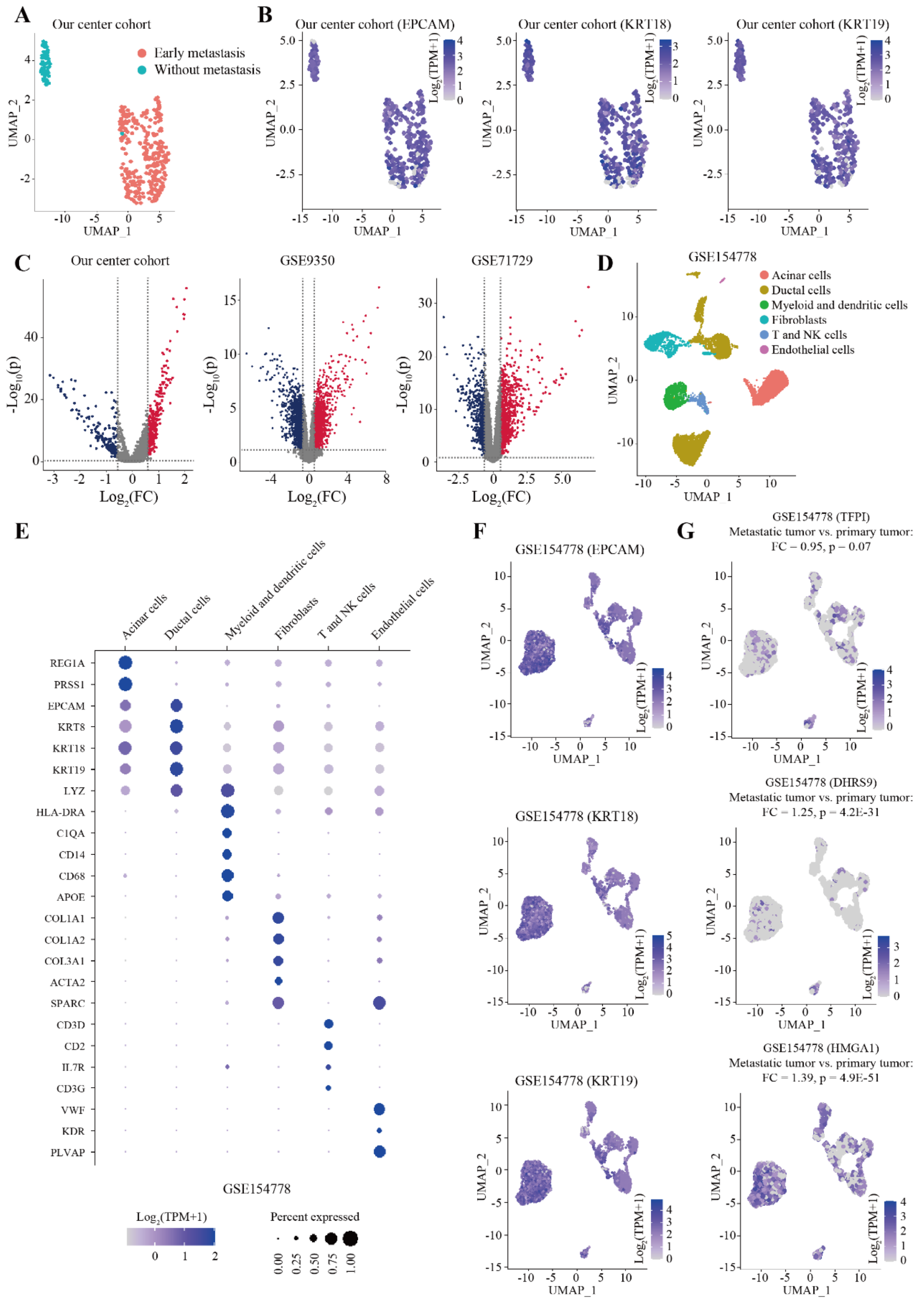

Figure S1. Bioinformatic analysis of potential target genes involved in PDAC metastasis. A) UMAP plot showing clusters of ductal cells from patients with different metastatic statuses in Tianjin Medical University Cancer Institute and Hospital (TJMUCIH). Our center cohort

represents the scRNA-seq of 2 PDAC patients with early metastasis (recurred within 6 months) for comparison with another 2 patients without metastasis more than 2 years after radical resection in TJMUCIH. B) UMAP plot showing the expression of marker genes in different clusters from patients in TJMUCIH (our center cohort). C) Volcano plots of differentially expressed genes (DEGs) in the three independent cohorts. GSE9350 is a microarray dataset for comparison of a high metastatic potential daughter cell line to its parental PDAC cell line; GSE71729 is another microarray dataset for comparative analysis between metastases and PDAC primary tumors. D) UMAP plot indicating different clusters of cell types from the GSE154778 dataset. GSE154778 is a public scRNA-seq dataset, representing a comparative analysis of the primary PDAC tumor and metastatic tumor. E) Bubble plot of cell type-specific markers of all clusters from the GSE154778 dataset. F) UMAP plot showing the expression of marker genes in different clusters from the GSE154778 dataset. G) UMAP plots analyzing the expression of TFPI, DHRS9, and HMGA1 in ductal cell clusters of PDAC primary tumors or metastases from the GSE154778 dataset.

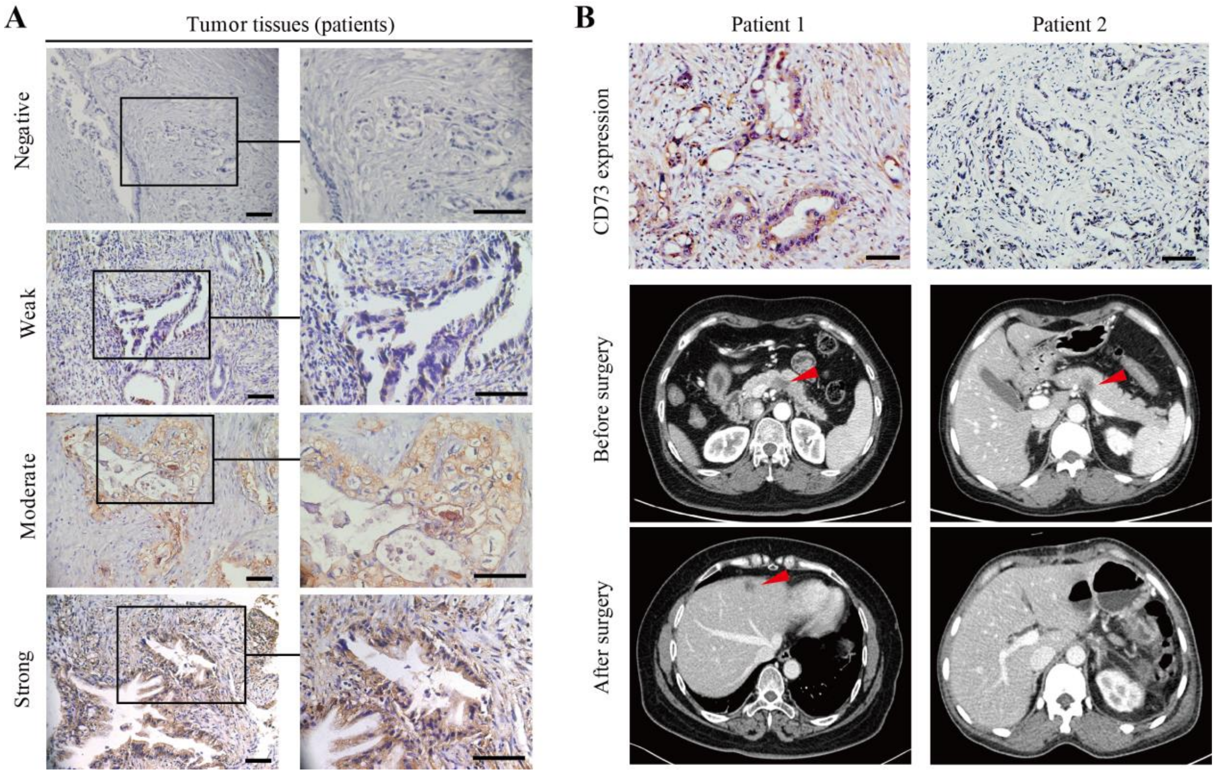

Figure S2. High expression of CD73 was associated with rapid PDAC metastasis. A) Representative pictures of indicated degree of CD73 expression in primary tumors of PDAC patients in IHC staining. Scale bar, 100 μm. B) Representative images of contrast-enhanced CT of patients with high or low expression of CD73. Patients with similar clinicopathological characteristics were followed up on the occurrence of liver metastasis within one year after radical resection. Arrowhead indicates primary or metastatic foci. Scale bar, 100 μm.

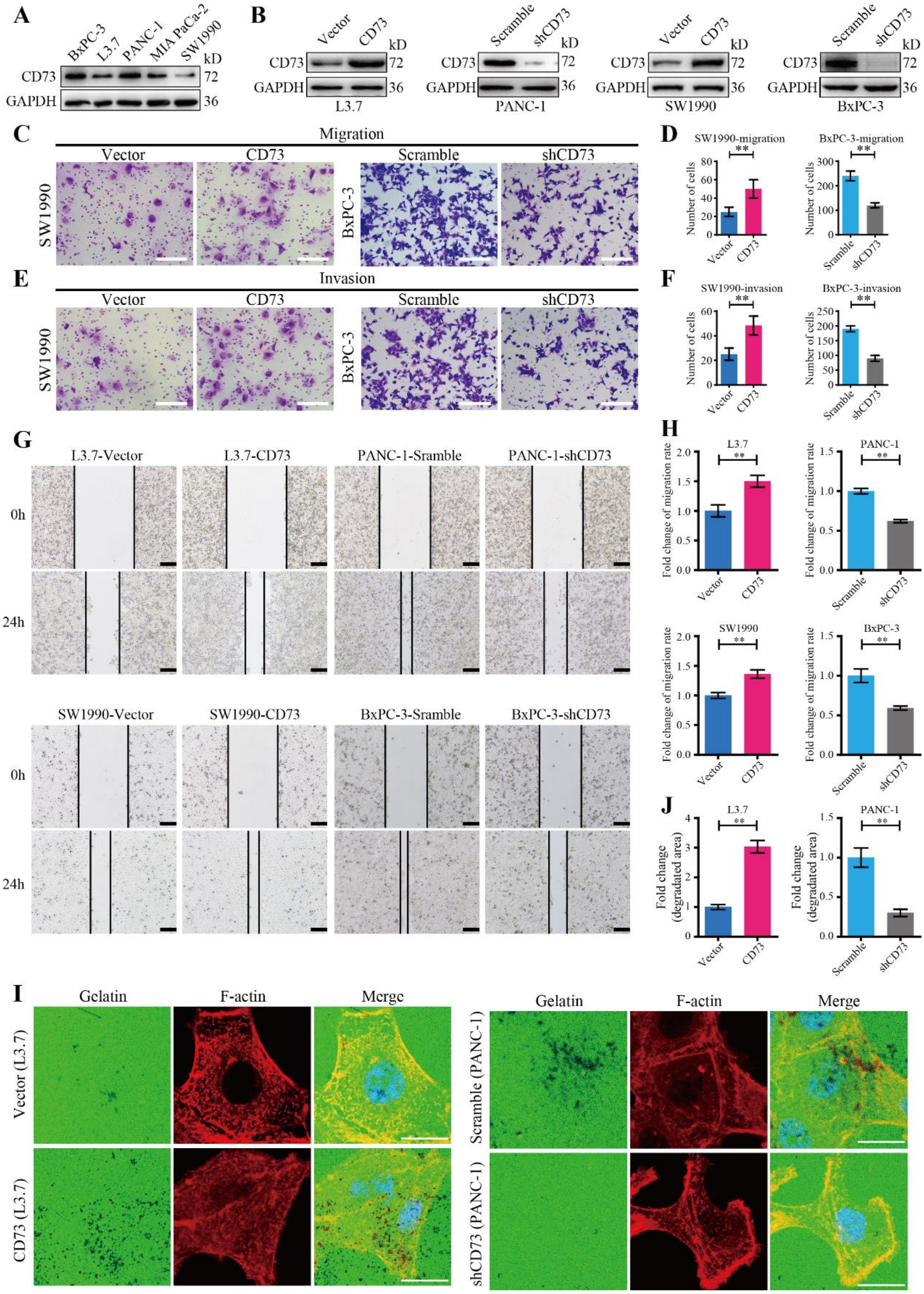

Figure S3. CD73 promotes migration and invasion of PDAC cells *in vitro*. A) Western blot analysis of endogenous CD73 in PDAC cell lines. B) L3.7 and SW1990 cells were transduced with CD73 or vector control, PANC-1 and BxPC-3 cells were transduced with CD73 shRNA or scramble control. Overexpression and knockdown efficacy of CD73 were determined by western blotting. C-F) Transwell assays detecting the migration (C-D) and invasion (E-F) of

SW1990 cells transduced with CD73 or vector control (Left), or BxPC-3 cells transduced with CD73 shRNA or scramble control (Right). Scale bar, 100 μm. G-H) Wound-healing assays determining the migration of L3.7 and SW1990 cells transduced with CD73 or vector control, or PANC-1 and BxPC-3 cells transduced with CD73 shRNA or scramble control. Representative images were shown in (G). Results of migration rate are presented as relative fold change after normalization to vehicle group (H). Scale bar, 200 μm. I-J) Gelatin degradation assays of L3.7 cells transduced with CD73 or vector control (Left), or PANC-1 cells transduced with CD73 shRNA or scramble control (Right). Representative images were shown in (I). Results of degradation area are presented as relative fold change after normalization to vehicle group (J). Scale bar, 10 μm. All values are means ± SD, n = 3. **, $p < 0.01$ in unpaired *t*-test. Data are representative of three independent experiments.

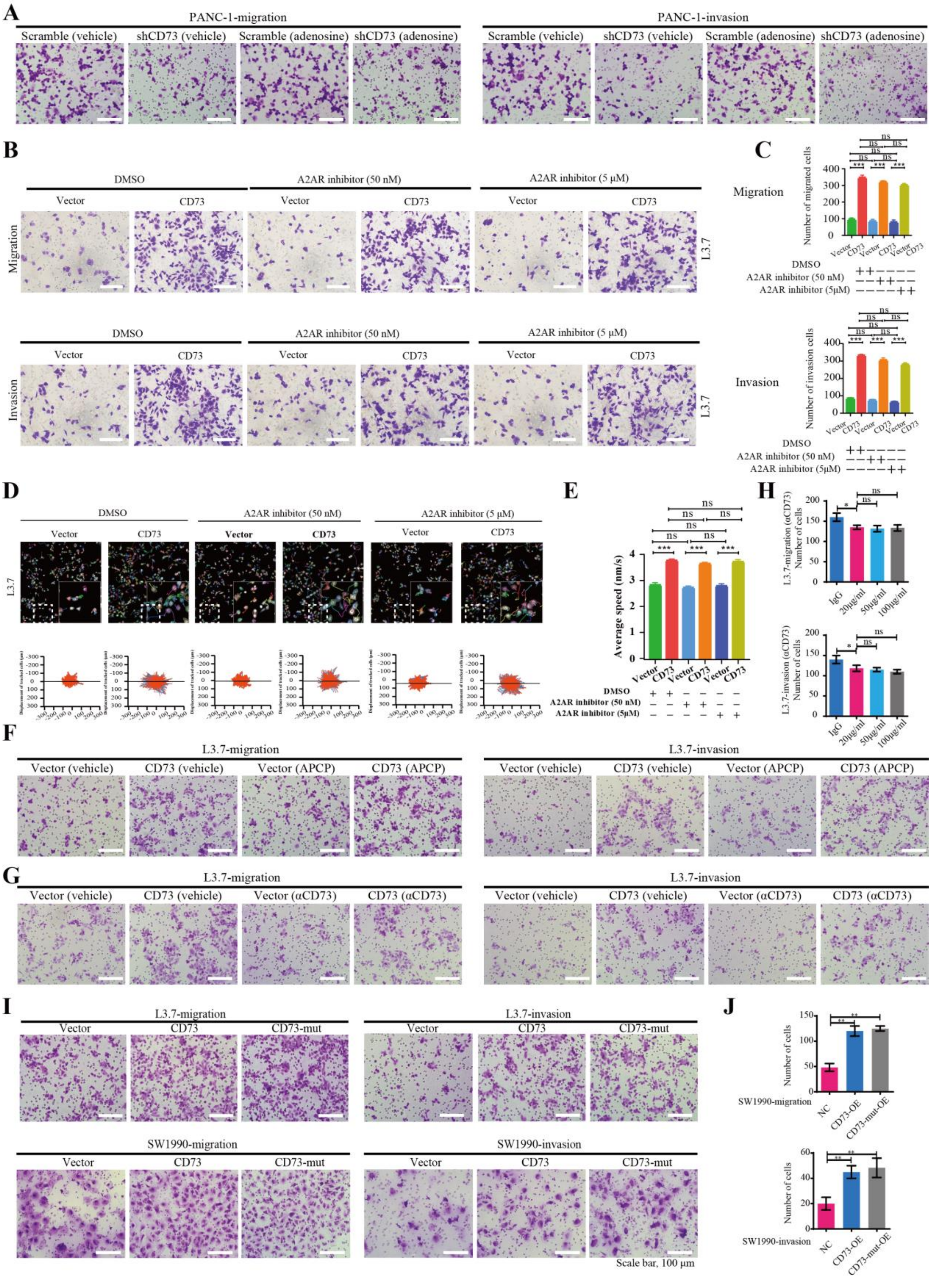

Figure S4. The nucleotidase activity is not required for CD73-induced migration and invasion in PDAC cells. A) Representative images of transwell assays examining the migration (Left) and invasion (Right) of PANC-1 cells transduced with CD73 shRNA or scramble control,

followed by treatment with 1 mM adenosine or vehicle. Scale bar, 100 μm. B-C) Representative images of (B) and quantifications of (C) migration (Up) and invasion (Down) of L3.7 cells transduced with CD73 or vector control, followed by treatment with 50 nM or 5 μM A2AR inhibitor SCH58261 or DMSO in transwell assays. Values are means ± SD, n = 3. ns, not significant, $p > 0.05$; ***, $p < 0.001$ in unpaired *t*-test. Scale bar, 100 μm. D-E) Representative images of (D) and quantifications of (E) cell movement analysis of L3.7 cells transduced with CD73 or vector control, followed by treatment with 50 nM or 5 μM SCH58261 or DMSO. Values are means ± SD, n = 3. ns, not significant, $p > 0.05$; ***, $p < 0.001$ in unpaired *t*-test. F-G) Representative images of migration (Left) and invasion (Right) of L3.7 cells transduced with CD73 or vector control, followed by treatment with 50 μM APCP (F) or 20 μg/ml αCD73 mAb (G) or vehicle in transwell assays. Scale bar, 100 μm. H) Quantifications of migration (Up) and invasion (Down) of L3.7 cells treated with different concentrations of αCD73 in transwell assays. I-J) Representative images of (I) and quantifications of (J) migration (Left) and invasion (Right) in L3.7 cells (Up) and SW1990 cells (Down) transduced with CD73, or CD73 mutant lacking catalytic activity (CD73-mut), or vector control in transwell assays. All values are means ± SD, n = 3. ns, not significant, $p > 0.05$; *, $p < 0.05$; **, $p < 0.01$ using one-way ANOVA. Data are representative of three independent experiments.

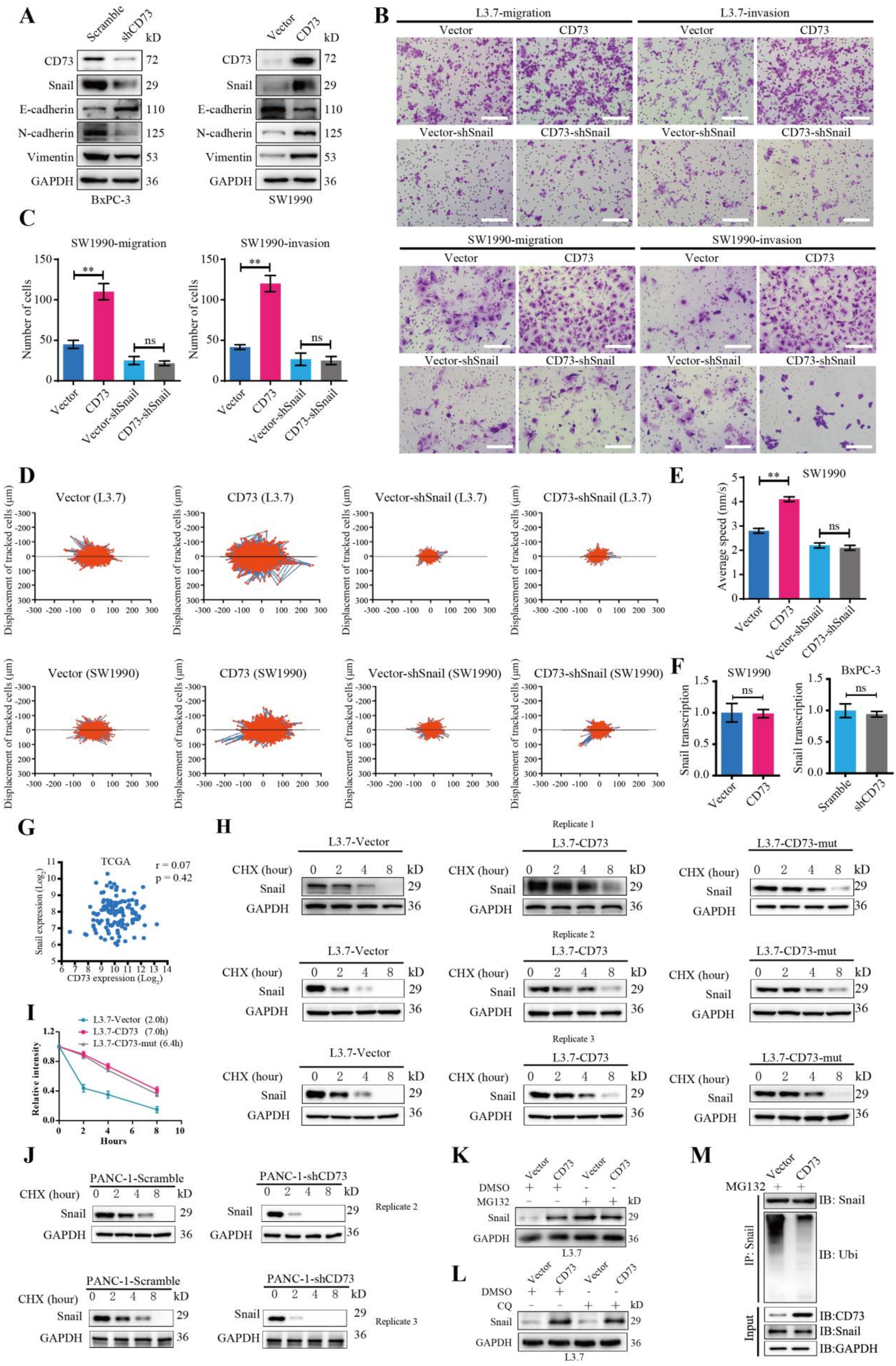
A
Scramble
shCD73
kD
CD73
Snail
E-cadherin
N-cadherin
Vimentin
GAPDH
BxPC-3
Vector
CD73
SW1990
B
L3.7-migration
L3.7-invasion
Vector-shSnail
CD73-shSnail
SW1990-migration
SW1990-invasion
C
Number of cells
D
Displacement of tracked cells (μm)
Vector (L3.7)
CD73 (L3.7)
Vector-shSnail (L3.7)
CD73-shSnail (L3.7)
Vector (SW1990)
CD73 (SW1990)
Vector-shSnail (SW1990)
CD73-shSnail (SW1990)
E
SW1990
Average speed (nm/s)
F
Snail transcription
Sramble
G
TCGA
r = 0.07
p = 0.42
Snail expression (Log2)
CD73 expression (Log2)
H
Replicate 1
Replicate 2
Replicate 3
L3.7-Vector
L3.7-CD73
L3.7-CD73-mut
CHX (hour)
I
L3.7-Vector (2.0h)
L3.7-CD73 (7.0h)
L3.7-CD73-mut (6.4h)
Relative intensity
Hours
J
PANC-1-Scramble
PANC-1-shCD73
K
DMSO
MG132
L3.7
L
CQ
M
IB: Snail
IP: Snail
IB: Ubi
IB:CD73
IB:Snail
IB:GAPDH
Input

Figure S5. CD73 promotes metastasis by upregulating Snail. A) Western blot analysis of Snail and EMT markers in BxPC-3 cells transduced with CD73 shRNA or scramble control (Left), or SW1990 cells transduced with CD73 or vector control (Right). B-E) Control or CD73 overexpressed L3.7 and SW1990 cells were transduced with Snail shRNA or scramble control respectively. Transwell (B, C) and cell movement (D, E) assays were performed to determine migration, invasion, and cell mobility of these cells respectively (n = 3). Values are means± SD. ns, not significant, $p > 0.05$; **, $p < 0.01$ using one-way ANOVA. Scale bar, 100 µm. F) qRT-PCR analysis of CD73 mRNA levels in SW1990 cells transduced with CD73 or vector control (Left), or BxPC3 cells transduced with CD73 shRNA or scramble control (Right). Data are presented as relative fold change after normalization to vehicle group. Values are means ± SD, n = 3. ns, not significant, $p > 0.05$ in unpaired *t*-test. G) Pearson correlation between CD73 and Snail at the mRNA level in TCGA database. H-I) L3.7 cells transduced with CD73, or CD73 mutant lacking catalytic activity (CD73-mut), or vector control were treated with 10 µg/ml cycloheximide (CHX) for indicated time.The degradation of the Snail protein was measured by western blot analysis. Representative images were shown in (H). The level of Snail was quantified after normalization to that of GAPDH using Image J (I). J) Representative images of western blot analysis of Snail in PANC-1 cells transduced with TRIM21 shRNA or scramble control, followed by treatment with 10 µg/ml CHX for indicated time, mesauring the degradation of the Snail protein. K-L) Western blot analysis of Snail in L3.7 cells transduced with CD73 or vector control, followed by treatment with MG132 (K) or chloroquine (CQ) (L) for 6h. M) L3.7 cells transduced with CD73 or vector control were trated with MG132 for 6h. Snail was immunoprecipitated and analyzed by western blotting to detect ubiquitylated Snail using an anti-ubiquitin antibody. Data are representative of three independent experiments.

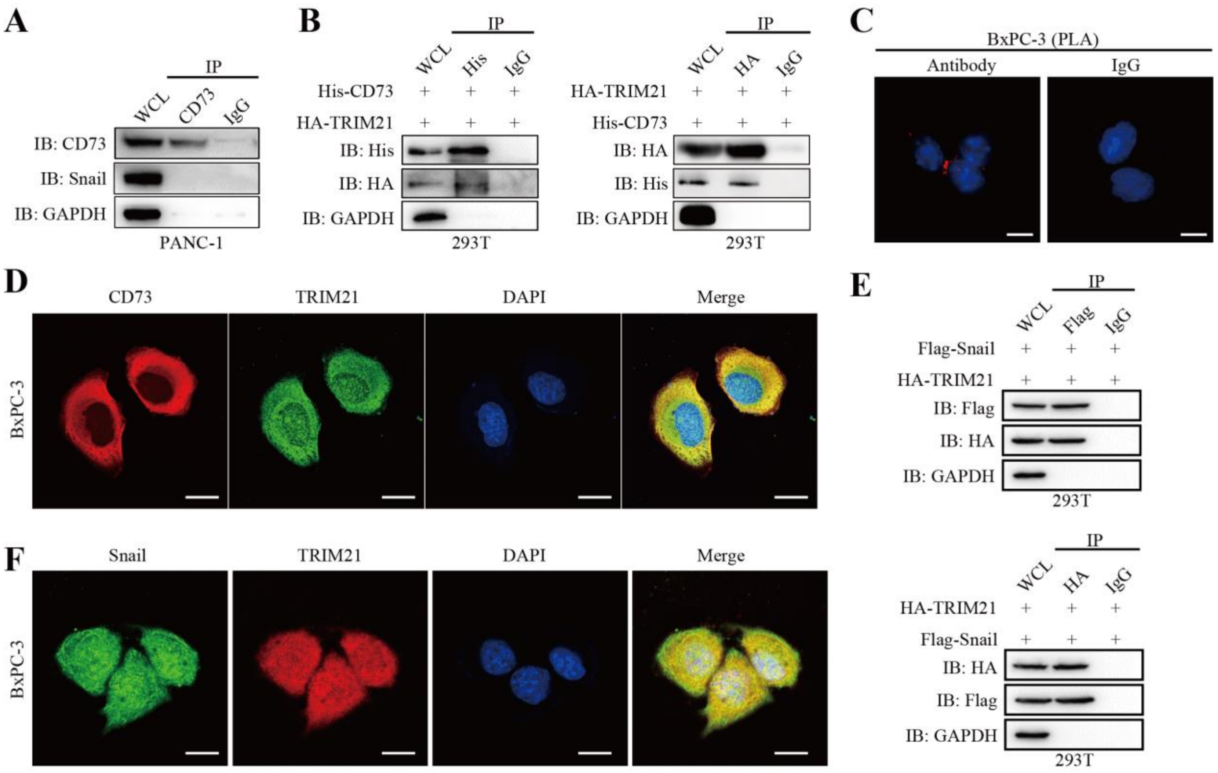

Figure S6. TRIM21 interacts with CD73 and Snail. A) Co-IP assays detecting the interaction between endogenous CD73 and Snail in PANC-1 cells. B) HEK-293T cells were co-transduced with His-CD73 and HA-TRIM21. Co-IP assays were performed using an anti-His or anti-HA antibodies to determine the interaction between exogenous CD73 and TRIM21. C) Proximity ligation assay (PLA) examining the binding between CD73 and TRIM21 in BxPC-3 cells *in vivo*. The red signal represents the formation of CD73-TRIM21 complex. Mouse IgG with same species and isotype was used as a negative control. Scale bar, 5 μm. D) Colocalization of CD73 (red) and TRIM21 (green) in the cytoplasm of BxPC-3 cells. Scale bar, 5μm. E) Co-IP assays showing the interaction between exogenous TRIM21 and Snail in BxPC-3 cells. F) Colocalization of Snail (green) and TRIM21 (red) in the cytoplasm of BxPC-3 cells. Scale bar, 5μm.

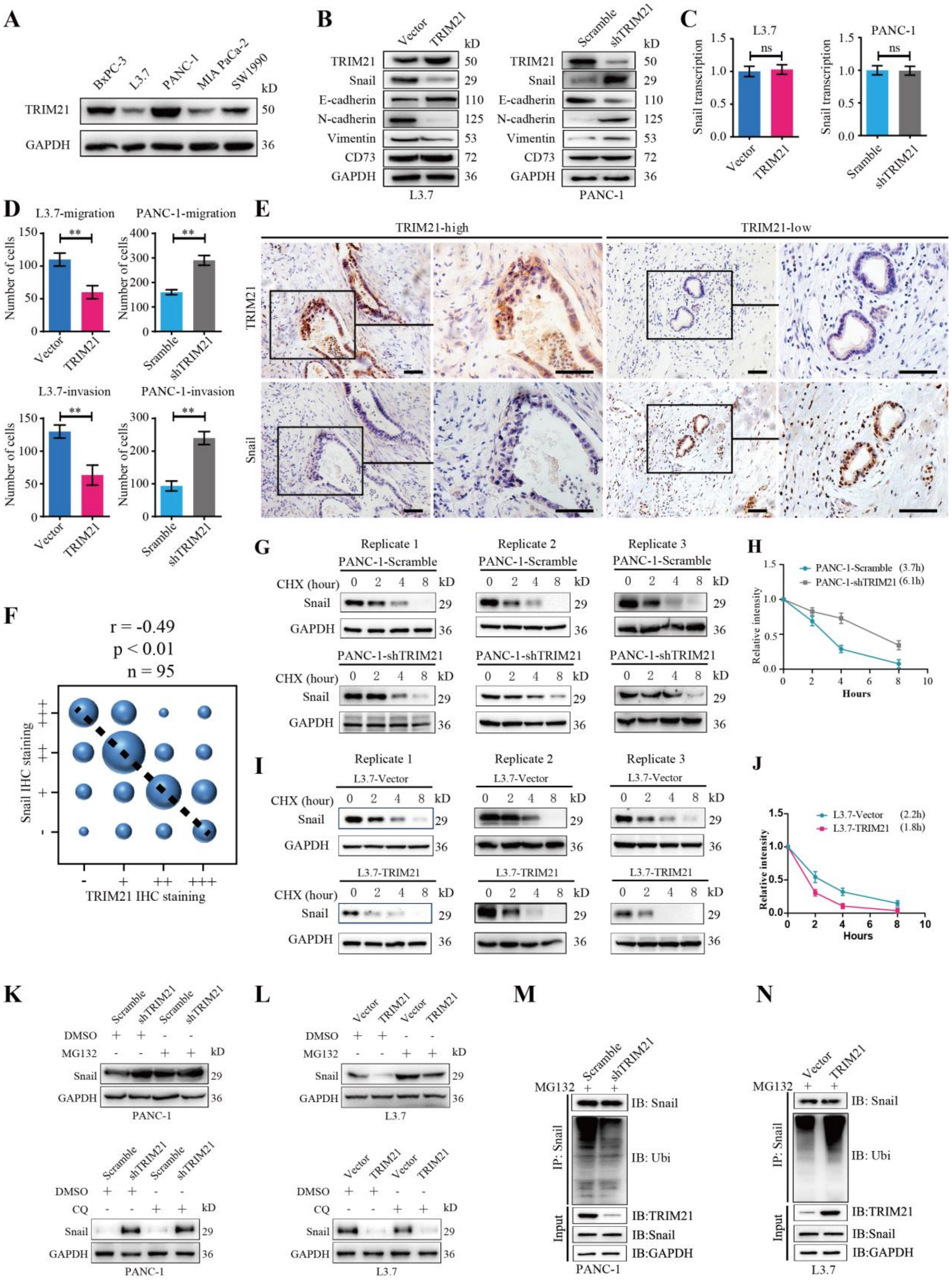

Figure S7. TRIM21 inhibits EMT by inducing ubiquitinoylation and degradation of Snail in PDAC cells. A) Western blot analysis of endogenous TRIM21 in muitiple PDAC cell lines. B) Western blot analysis of TRIM21 and EMT markers in L3.7 cells transduced with TRIM21 or vector control (Left), or PANC-1 cells transduced with TRIM21 shRNA or scramble

control (Right). C) qRT-PCR analysis of Snail mRNA levels in L3.7 cells transduced with CD73 or vector control (Left), or PANC-1 cells transduced with CD73 shRNA or scramble control (Right). Data are presented as relative fold change after normalization to vehicle group. Values are means ± SD, n = 3. ns, not significant, $p > 0.05$ in unpaired *t*-test. D) Transwell assays detecting the migration (Up) and invasion (Down) of L3.7 cells transduced with TRIM21 or vector control (Left), or PANC-1 cells transduced with Trim21 shRNA or scramble control (Right). Values are means ± SD, n = 3. ns, not significant, $p > 0.05$; **, $p < 0.01$ using one-way ANOVA. E-F) Representative IHC staining of (E) and spearman rank correlation of (F) TRIM21 and Snail in PDAC tissues. The bubble size represents the number of patients with indicated staining degree. Scale bar, 100 μm. G-J) PANC-1 cells transduced with TRIM21 shRNA or scramble control (G-H), or L3.7 cells transduced with TRIM21 or vector control (I-J) were treated with 10 μg/mL cycloheximide (CHX) for indicated times. The degradation of the Snail protein was measured by western blot analysis. Representative images were shown in (G and I). The level of Snail was quantified after normalization to that of GAPDH using Image J (H and J). K-L) Western blot analysis of Snail in PANC-1 cells transduced with TRIM21 shRNA or scramble control (K), or L3.7 cells transduced with TRIM21 or vector control (L), followed by treatment with MG132 (Up) or chloroquine (CQ) (Down) for 6h. M-N) PANC-1 cells transduced with TRIM21 shRNA or scramble control (M), or L3.7 cells transduced with TRIM21 or vector control (N) were trated with MG132 for 6h. Snail was immunoprecipitated and analyzed by western blotting to detect ubiquitylated Snail using an anti-ubiquitin antibody. Data are representative of three independent experiments.

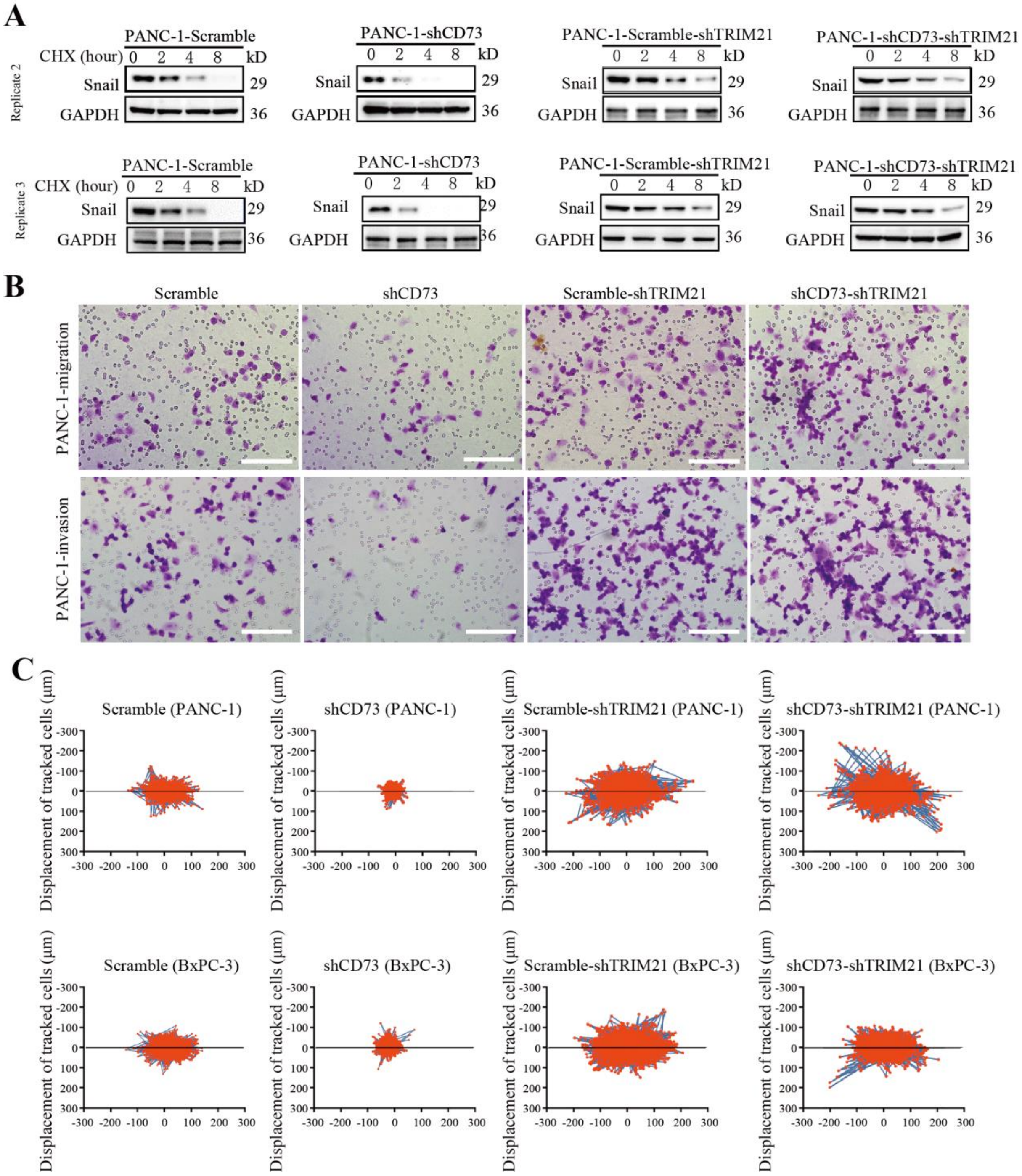

Figure S8. TRIM21 is indispensable for the metastatic property of CD73. Control or CD73 knockdown PANC-1 cells and BxPC-3 were transduced with TRIM21 shRNA or scramble control respectively. A) Representative images of western blot analysis of Snail in PANC-1 cells treated with 10 μg/mL cycloheximide (CHX) for indicated times, mseauring the degradation of Snail protein. B) Representative images of migration (Up) and invasion (Down) of PANC-1 cells with indicated genotypes in transwell assays. Scale bar, 100μm. C) Representative dynamic images of PANC-1 (Up) and BxPC-3 (Down) cells with indicated genotypes. Data are representative of three independent experiments.

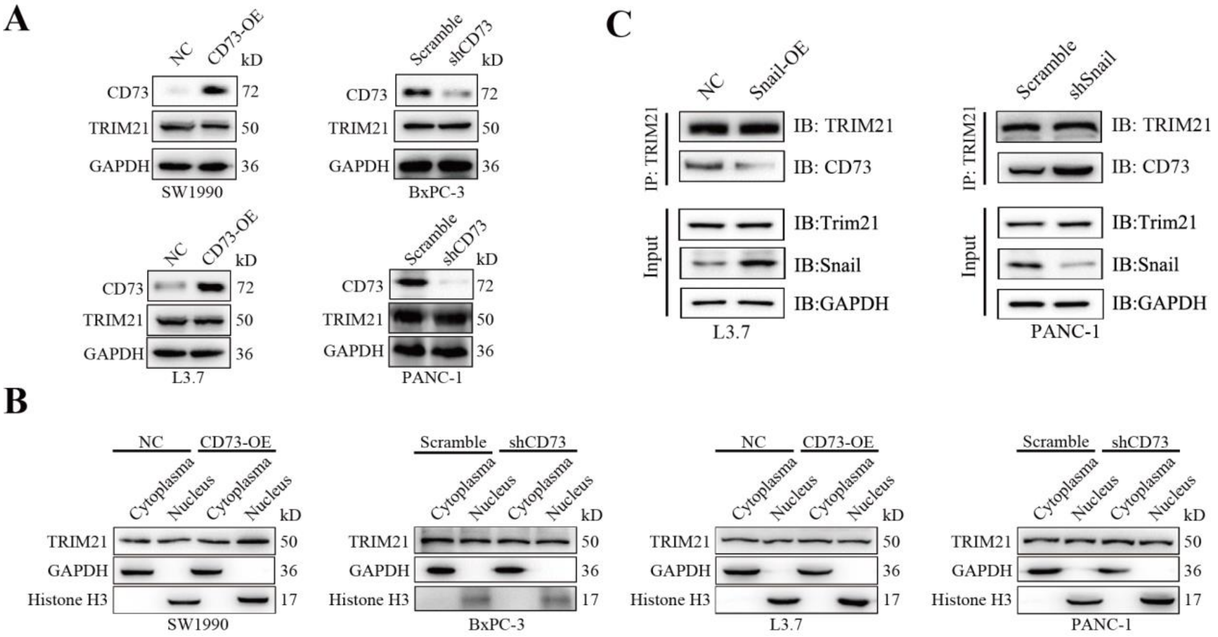

Figure S9. CD73 doesn't affect the protein level and subcellular localization of TRIM21 in PDAC cells. A) Western blot analysis of TRIM21 in SW1990 and L3.7 cells transduced with CD73 or vector control, or BxPC-3 and PANC-1 cells transduced with CD73 shRNA or scramble control. B) Cytoplasmic protein and nuclear protein of SW1990 and L3.7 cells transduced with CD73 or vector control, or BxPC-3 and PANC-1 cells transduced with CD73 shRNA or scramble control were collected using a commercial kit. The distributions of TRIM21 were analyzed by western blot analysis. GAPDH and Histone 3 (H3) were used as loading control for cytoplasmic protein and nuclear protein respectively. C) TRIM21 was immunoprecipitated from L3.7 cells transduced with Snail or vector control (Left), or PANC-1 cells transduced with Snail shRNA or scramble control (Right), and analyzed by western blotting to detect the binding of endogenous TRIM21 to CD73. Data are representative of three independent experiments.

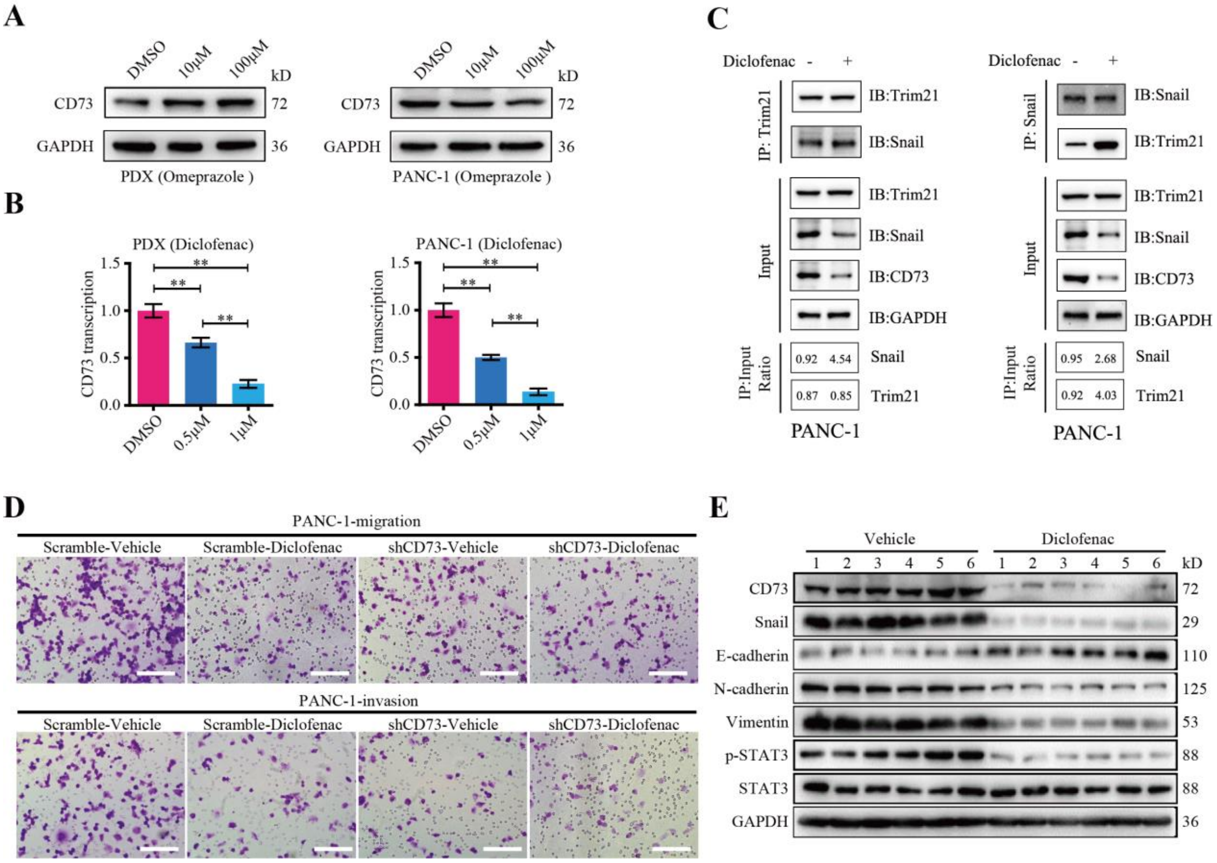

Figure S10. Diclofenac inhibits the expression of CD73 and suppresses EMT, migration and invasion of PDAC cells. A) Western blot analysis of CD73 in PDX derived cells (Left) and PANC-1 cells (Right) treated with different concentrations of omeprazole. B) qRT-PCR analysis of CD73 mRNA levels in PDX derived cells (Left) and PANC-1 cells (Right) treated with 0.5 or 1 μM diclofenac, or vehicle. qRT-PCR data are presented as relative fold change after normalization to vehicle group. Values are means ± SD, n = 3. **, $p < 0.01$ using one-way ANOVA. C) TRIM21 and Snail were immunoprecipitated from PANC-1 cells treated with 1 μM diclofenac or vehicle, and analyzed by western blotting to detect the the binding between TRIM21 and Snail. The levels of Snail and TRIM21 were measured by Image J. The IP:Input ratio was caculated by normalizing the value of immunoprecipitate group to that of input group. D) Representative images of migration (Up) and invasion (Down) of PANC-1 cells transduced with CD73 shRNA or scramble control, followed by treatment with 1 μM diclofenac or vehicle. Scale bar, 100 μm. E) Western blot analysis of CD73, Snail, EMT markers (E-cadherin, N-cadherin, and Vimentin) and STAT3 in tumor xenografts of PANC-1 cells treated intraperitoneal daily with 20 mg/kg of diclofenac or vehicle. Data are representative of three independent experiments.

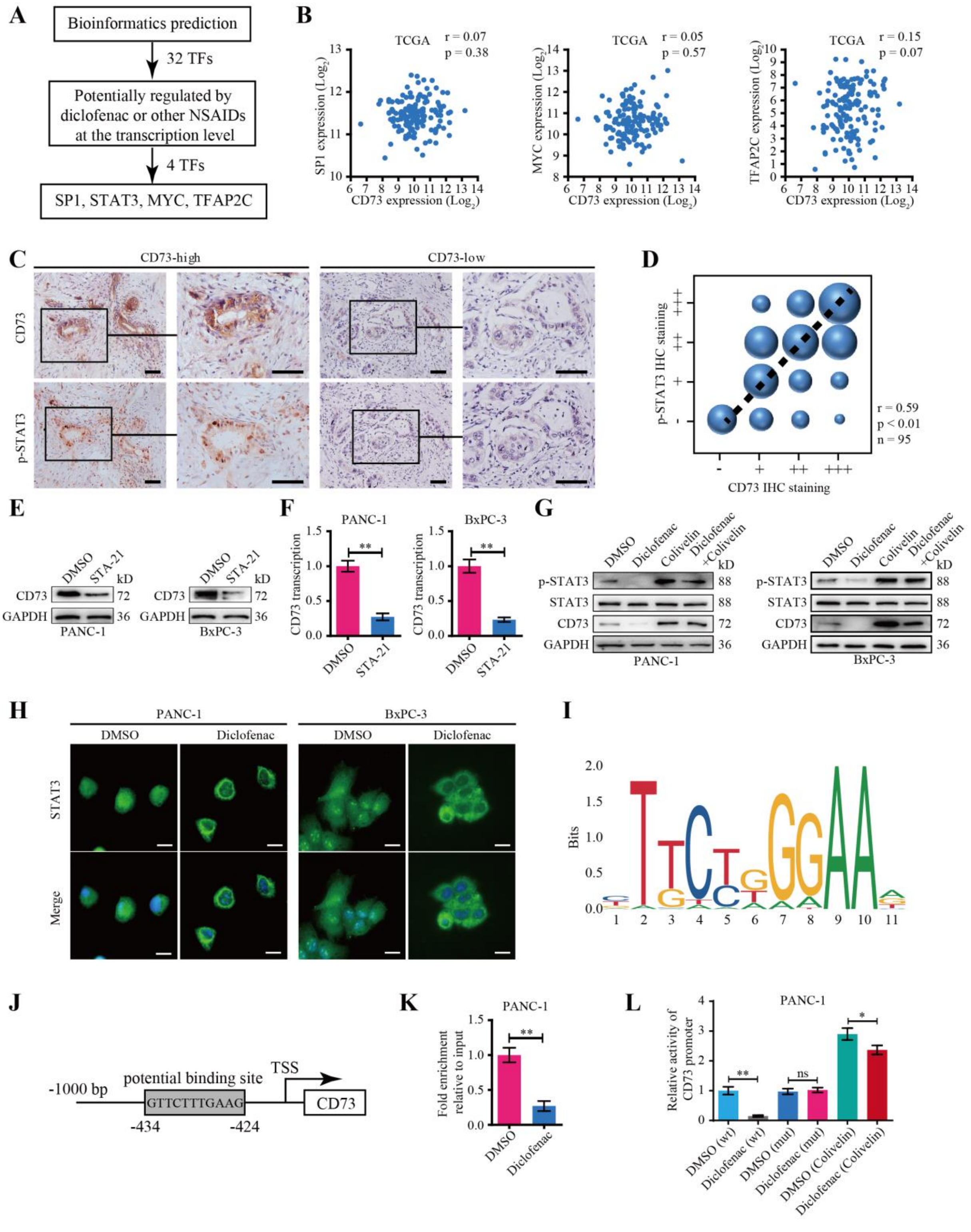

Figure S11. Diclofenac inhibits the expression of CD73 partially via inactivating STAT3. A) Schematic for screening the downstream transcription factors (TFs) of diclofenac that potentially regulate the transcription of CD73. B) Pearson correlation between CD73 and indicated transcription factor in TCGA database. C-D) Representative IHC staining of (C) and spearman correlation analysis of (D) CD73 and p-STAT3 in PDAC tissues. The bubble size represents the number of patients with indicated staining degree. Scale bar, 100 μm. E-F)

Western blot (E) and qRT-PCR (F) analysis of CD73 protein and mRNA levels in PANC-1 (Left) and BxPC-3 (Right) cells treated with 10 μM STAT3 inhibitor STA-21 or DMSO. Values are means ± SD, n = 3. **, $p < 0.01$ in unpaired *t*-test. G) Western blot analysis of CD73, STAT3 and p-STAT3 in PANC-1 (Left) and BxPC-3 (Right) cells treated with 1 μM diclofenac, or 1 μM STAT3 agonist colivelin, or together, or vehicle. H) Representative immunofluorescence staining of STAT3 in PANC-1 (Left) and BxPC-3 (Right) cells treated with 1 μM diclofenac or vehicle. Scale bar, 5 μm. I) The in known binding motif of STAT3. J) Predicted binding site of STAT3 on the promoter of human CD73. K) ChIP-qPCR analysis determining the binding of STAT3 on the CD73 promoter in PANC-1 cells treated with 1 μM diclofenac or vehicle. ChIP-qPCR data were quantified using a Percent Input Method and then normalized to vehicle group. Values are means ± SD, n = 3. **, $p < 0.01$ in unpaired *t*-test. L) PANC-1 cells were transfected with recombinant pGL3.0 reporter vector containing wild-type (wt) or STAT3 binding site deficient (mut) CD73 promoter respectively, and treated with 1 μM diclofenac, or 1 μM colivelin, or together, or vehicle. The activity of CD73 promoters were determined by standard dual-luciferase assay. Firefly luciferase activities were normalized to that of Renilla luciferase. Results are presented as relative fold change after normalization to vehicle group in the first lane. Values are means ± SD, n = 3. ns, not significant, $p > 0.05$; *, $p < 0.05$, **, $p < 0.01$ in unpaired *t*-test. Data are representative of three independent experiments.

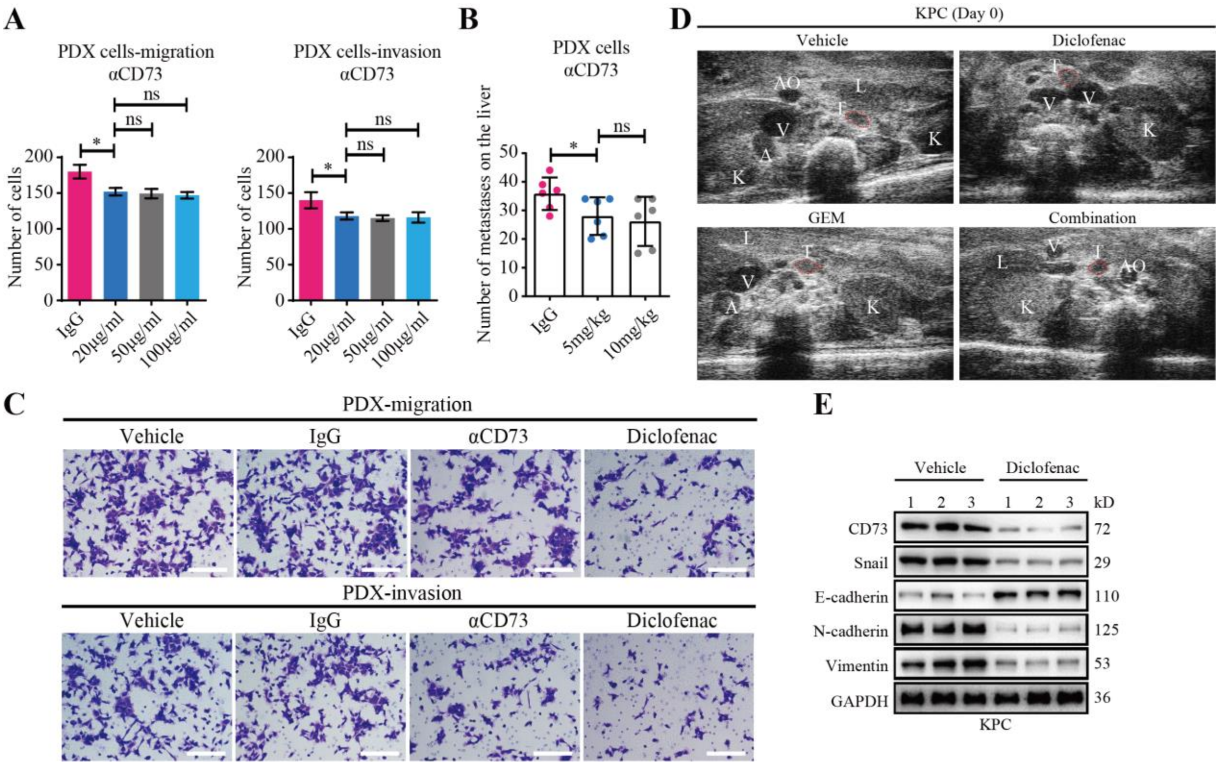

Figure S12. αCD73 mAb mildly suppresses migration and invasion of PDAC cells, diclofenac

has great potential to improve the therapeutic outcome of gemcitabine for treatment of metastatic PDAC in KPC mice models. A) Migration and invasion of PDX derived cells treated with different concentrations of αCD73 mAb in transwell assays, determining the optimal dose of αCD73 mAb in cell culture. Values are means ± SD, n = 3. ns, not significant, $p > 0.05$; *, $p < 0.05$ using one-way ANOVA. B) Nude mice were orthotopically injected with PDX-derived cells, and treated intravenous with 5 or 10 mg/kg of αCD73 mAb, or normal IgG control every 3 days starting on day 4. Number of metastatic foci on the appearance of liver were measured and quantified on day 28. Values are means ± SD, n = 6. ns, not significant, $p > 0.05$; *, $p < 0.05$ using one-way ANOVA. C) Representative images of migration (Up) and invasion (Down) of PDX derived cells treated with 20 μg/mL αCD73 mAb or normal IgG, or 1 μM diclofenac, or vehicle for 48 hours in transwell assays. Scale bar, 100 μm. D) Representative ultrasound images of KPC mice at the time of grouping. Red dashed circles mark the primary PDACs. A, adrenal gland; AO, aorta; L, liver; K, kidney; T, tumor; V, vein. E) Two-month-old KPC mice in (D) were treated intraperitoneal daily with 20 mg/kg of diclofenac or vehicle. Tumor tissues were collected at day 90. CD73, Snail and EMT markers (E-cadherin, N-cadherin, and Vimentin) were detected by western blot analysis. Data are representative of three independent experiments.

Supplementary Tables

Table S1. Patient characteristics.

**Characteristics**	**High expression**	**Low expression**	***p* value**
Number of patients	8	8	-
Radical surgery			
Yes/no	8/0	8/0	1.00
Standard adjuvant chemotherapy			
Yes/no	8/0	8/0	1.00
Age	60 ± 5	63 ± 6	0.89
Gender			
Male/female	5/3	5/3	1.00
Diameter of tumor (cm)	3.0 ± 1.3	3.0 ± 1.2	0.98
Lymph node metastasis			
Yes/no	2/6	2/6	1.00
Pathological stage			
I/II/III	5/1/2	5/1/2	1.00
Differentiation			
Poor/moderate	4/4	4/4	1.00
Primary tumor location			
Head/body	6/2	6/2	1.00
Liver metastasis within one year			
Yes/no	5/3	0/8	0.03

PDAC patients with similar clinicopathological characteristics between the two groups were prospectively included in the analysis. The incidence of liver metastasis significantly increased in patients with a high expression level of CD73 during the follow-up period.

Table S2. Antibodies used in this study.

**Antibody**	**Supplier**	**Catalog no.**	**Usage**
CD73	Abcam	ab175396	WB: 1: 1000<br>IHC: 1: 300<br>IF: 1: 200
CD73	Santa Cruz Biotechnology	sc-32299	IP: 1 μg/test
CD73	Biolegend	344002	Treatment: indicated concentration
CK19	Abcam	ab52625	IHC: 1: 300
Snail	Santa Cruz Biotechnology	sc-271977	WB: 1: 1000<br>IHC: 1: 100<br>IF: 1: 200
Snail	CST	3879	IP: 1: 50
Snail	Proteintech	13099-1-AP	IF: 1: 100
E-cadherin	Abcam	ab1416	WB: 1: 1000
N-cadherin	Abcam	ab18203	WB: 1: 1000
Vimentin	Abcam	ab92547	WB: 1: 1000
GAPDH	Santa Cruz Biotechnology	sc-47724	WB: 1:5000
Ubiquitin	Abcam	ab7254	WB: 1: 1000
TRIM21	Santa Cruz Biotechnology	sc-25351	IP: 1 μg/test<br>WB: 1: 1000<br>IF: 1: 50<br>IHC: 1: 100
STAT3	CST	9139	WB: 1: 1000<br>ChIP: 1:100
p-STAT3 (Y705)	Abcam	ab171358	WB: 1: 1000<br>IHC: 1: 100
Flag	Sigma-Aldrich	F1804	WB: 1: 1000<br>IP: 1 μg/test
HA	CST	3724	WB: 1: 1000<br>IP: 1: 50
GST	Santa Cruz Biotechnology	sc-138	WB: 1: 1000
His	Santa Cruz Biotechnology	sc-8036	WB: 1: 1000
Histone H3	CST	4499	WB: 1:2000
Normal IgG	Santa Cruz Biotechnology	sc-2025	IP: 1 μg/test<br>Treatment: indicated concentration

Table S3. Primers and oligonucleotides sequences.

**Primer name**		**Sequences**
PCR primers		
Snail	Forward	CCAATCGGAAGCCTAACTA
	Reverse	AAGGAAGAGACTGAAGTAGAG
CD73	Forward	TCGGCTCTTCACCAAGGTTCAG
	Reverse	GGCTCGATCAGTCCTTCCACAC
ACTB	Forward	CTACCTTCAACTCCATCATGAAGTG
	Reverse	TGCGCTCAGGAGGAGC
CHIP primers		
STAT3 binding site on the promoter of human CD73		
Forward		AACAGGAAGCGTCACATC
Reverse		GCGAGGAAGGAATTGGAG
Interfering oligonucleotides		
shCD73		GCCTGGGAGCTTACGATTTATGGATCCATAAATCGTAAGCTCCCAGGC
shSnail		GCTCTTTCCTCGTCAGGAATTGGATCCAATTCCTGACGAGGAAAGAGC
shTRIM21		GGAATGCATCTCTCAGGTTTTGGATCCAAAACCTGAGAGATGCATTCC

Table S4. Main reagents

Reagents	Supplier	Catalog no.	Usage
Cycloheximide	Selleck	S7418	10μg/ml
MG132	MCE	HY-13259	10μM
CQ	MCE	HY-17589A	10μM
Adenosine	Sigma	A9251	1mM
APCP	Sigma	M3763	50μM
GST	Sino Biological	11213-HNAE	-
GST-TRIM21	Abnova	H00006737-P01	-
HA-TRIM21	Dia-An Biotechnology	-	-
His-CD73	Dia-An Biotechnology	-	-
Flag-Snail	Dia-An Biotechnology	-	-
Omeprazole	MCE	HY-B0113	Indicated concentration
Diclofenac	MCE	HY-15036	Indicated concentration
GEM	Selleck	S1714	20mg/body weight (kg)
STA-21	MCE	HY-18061	10μM
Colivelin	MCE	HY-P1061	1μM
NE-PER™ Nuclear and Cytoplasmic Extraction Reagents	ThermoFisher	78833	-
QCM™ Gelatin Invadopodia Assay (Green)	Sigma-Aldrich	ECM670	-
SCH58261	MCE	HY-19533	Indicated concentration

Supplementary Materials and Methods

Chromatin immunoprecipitation (ChIP) and Dual-luciferase assay

ChIP was conducted using the EZ-ChIP kit according to the manufacturer's instructions (Millipore, USA) and then subjected to qPCR assay following Percent Input Method. The PCR primers are listed in **Table S2**.

For Dual-luciferase assay, PANC-1 cells were transfected with recombinant pGL3.0 reporter vector containing wild-type or STAT3 binding site deficient CD73 promoter respectively, and treated with 1 μM diclofenac, or 1 μM colivelin, or together, or vehicle in 24-well plates. Lysates were prepared 48 hours after transfection, and luciferase activities were measured using the Dual-luciferase Reporter Assay System (Promega, USA according to the manufacturer's protocols. Firefly luciferase activities were normalized to that of Renilla luciferase.
